# Inhibition of HIV-1 endocytosis allows lipid mixing at the plasma membrane, but not complete fusion

**DOI:** 10.1186/1742-4690-8-99

**Published:** 2011-12-06

**Authors:** Michelle de la Vega, Mariana Marin, Naoyuki Kondo, Kosuke Miyauchi, Yuri Kim, Raquel F Epand, Richard M Epand, Gregory B Melikyan

**Affiliations:** 1 Division of Pediatric Infectious Diseases, Emory University Children's Center, 2015 Uppergate Drive, Atlanta, GA 30322; 2 RIKEN Yokohama Institute, 1-7-22 Suehiro-cho, Tsurumi-ku, Yokohama City, Kanagawa, 230-0045, Japan; 3 Department of Neuroscience, Georgetown University, 3900 Reservoir Road, Washington DC 20007; 4 McMaster University, Dept. Biochemistry and Biomedical Sciences, Health Sciences Centre 1280 Main Street West, Hamilton ON L8S 4K1, Canada; 5 Children's Healthcare of Atlanta, Atlanta, GA 30322, USA

**Keywords:** HIV fusion kinetics, hemifusion, fusion pore, dynasore, temperature-arrested intermediate, fusion inhibitors, single particle tracking, beta-lactamase, intrinsic membrane curvature

## Abstract

**Background:**

We recently provided evidence that HIV-1 enters HeLa-derived TZM-bl and lymphoid CEMss cells by fusing with endosomes, whereas its fusion with the plasma membrane does not proceed beyond the lipid mixing step. The mechanism of restriction of HIV-1 fusion at the cell surface and/or the factors that aid the virus entry from endosomes remain unclear.

**Results:**

We examined HIV-1 fusion with a panel of target cells lines and with primary CD4^+ ^T cells. Kinetic measurements of fusion combined with time-resolved imaging of single viruses further reinforced the notion that HIV-1 enters the cells *via *endocytosis and fusion with endosomes. Furthermore, we attempted to deliberately redirect virus fusion to the plasma membrane, using two experimental strategies. First, the fusion reaction was synchronized by pre-incubating the viruses with cells at reduced temperature to allow CD4 and coreceptors engagement, but not the virus uptake or fusion. Subsequent shift to a physiological temperature triggered accelerated virus uptake followed by entry from endosomes, but did not permit fusion at the cell surface. Second, blocking HIV-1 endocytosis by a small-molecule dynamin inhibitor, dynasore, resulted in transfer of viral lipids to the plasma membrane without any detectable release of the viral content into the cytosol. We also found that a higher concentration of dynasore is required to block the HIV-endosome fusion compared to virus internalization.

**Conclusions:**

Our results further support the notion that HIV-1 enters disparate cell types through fusion with endosomes. The block of HIV-1 fusion with the plasma membrane at a post-lipid mixing stage shows that this membrane is not conducive to fusion pore formation and/or enlargement. The ability of dynasore to interfere with the virus-endosome fusion suggests that dynamin could be involved in two distinct steps of HIV-1 entry - endocytosis and fusion within intracellular compartments.

## Background

Infection of cells by human immunodeficiency virus type 1 (HIV-1) is a multistep process beginning with the sequential binding of the gp120 subunit of the viral envelope glycoprotein (Env) to CD4 and a coreceptor CCR5 or CXCR4 [1,2]. Interactions with CD4 and coreceptor trigger conformational changes in the transmembrane subunit of Env, gp41, which ultimately mediates membrane fusion [3,4]. As with other viruses that do not depend on low pH for entry, HIV-1 has been widely believed to undergo fusion at the plasma membrane, whereas endocytosis has been regarded as a nonproductive pathway leading to virus degradation (for example, [5-7]). This view is based mainly on the following lines of evidence. First, HIV-1 Env mediates cell-cell fusion at neutral pH [7,8], and the virus itself can fuse adjacent cells expressing CD4 and coreceptors [9,10] (termed "fusion from without"). Second, HIV-1 infection is not compromised by mutations in the cytoplasmic domains of CD4 or coreceptors that severely impair their ability to undergo ligand-mediated endocytosis [5,6,11,12]. Third, in contrast to HIV-1, VSV G-pseudotyped HIV particles, which constitutively enter through endocytosis, exhibit different requirements for HIV-1 accessory proteins for infection [13], and strikingly, fail to infect resting CD4^+ ^T cells [14-16]. Also, the membrane transport activity of Arf6 (ADP-ribosylation factor 6) appears essential for clathrin-independent CD4/HIV-1 co-internalization and fusion, but not for fusion of VSV G pseudotypes [17].

The above evidence, while supporting HIV-1 fusion with the plasma membrane, are somewhat indirect and generally do not rule out the existence of an endocytic entry pathway for this virus. For instance, the lack of low pH-dependence [8,18,19] simply means that HIV-1 fusion is not restricted to acidic compartments. It also remains to be demonstrated that CD4 and coreceptor mutants impaired in ligand-mediated endocytosis do not co-internalize with the virus, which would allow fusion with endosomes. On the other hand, accumulating evidence support the existence of productive HIV-1 entry through endocytosis. The observation that *trans-*dominant negative mutants of dynamin-2 and Eps15 potently inhibit HIV-1 fusion and infection [20] implies that this virus relies, at least in part, on clathrin-mediated endocytosis for productive entry. Moreover, a specific small-molecule inhibitor of clathrin function interferes with HIV-1 uptake and infectivity [21]. Finally, inhibition of dynamin GTPase activity by dynasore effectively suppressed clathrin-dependent uptake of transferrin and low density lipoprotein [22], as well as HIV-1 endocytosis, fusion and infectivity [23].

By employing non-invasive imaging technologies and functional assays, we have gained further insights into the mechanism of HIV-1 entry [23]. First, visualization of single virus entry into cells revealed two types of fusion events - transfer of the viral lipids into the plasma membrane without the subsequent release of the viral content (hemifusion) and release of the viral content without significant dilution of the viral membrane marker, which corresponds to complete virus fusion with small intracellular compartments. Second, comparison of the rates of HIV-1 escape from a membrane-impermeable fusion inhibitor and from low temperature applied at different times during the virus entry demonstrated a delayed protection from the temperature block compared to resistance to a fusion inhibitor. Collectively, these findings imply that HIV-1 fuses with endosomes, but fails to undergo complete fusion with the plasma membrane. We also found that endosomal fusion was inhibited by a dynamin-2 inhibitor, dynasore, suggesting that dynamin is involved both in the virus uptake and in the subsequent fusion step occurring within endosomes [23].

The basis for HIV's strong preference for entry through endosomes is not clear. Contrary to the model proposed in [24], kinetic measurements of lipid mixing with the plasma membrane demonstrated that the lack of complete fusion at the cell surface was not due to the faster virus uptake which would clear the virus from the plasma membrane before it undergoes fusion. Nearly 80% of Env- and receptor-dependent lipid mixing events at the plasma membrane occurred before significant endocytosis or endosomal fusion were detected [23,25]. Here, to gain further insight into the determinants of HIV-1 fusion, we tested whether this virus was able to fuse with the plasma membrane of distinct target cells, such as lymphoid cells and U87.CD4.CCR5 cells, which appeared to support limited fusion between immobilized viruses and the plasma membrane of detached cells [26]. Moreover, we attempted to redirect HIV-1 fusion to the cell surface by preventing virus uptake, using endocytosis inhibitors or reduced temperature. However, these interventions did not favor complete fusion at the cell surface, in spite of the extended window of opportunity to enter from the plasma membrane. Our results thus reinforce the notion that HIV-1 enters different cell types by endocytosis and demonstrate that the failure of this virus to fuse with the plasma membrane is not due to the kinetically dominant endocytosis that removes fusogenic viruses from the cell surface. Finally, we found that dynasore blocked productive endocytosis of HIV-1 more efficiently than virus-endosome fusion, consistent with a more critical role of dynamin in endocytic vesicle formation compared to virus-endosome fusion.

## Results

### A functional approach to define the virus entry pathways

**Figure 1:**
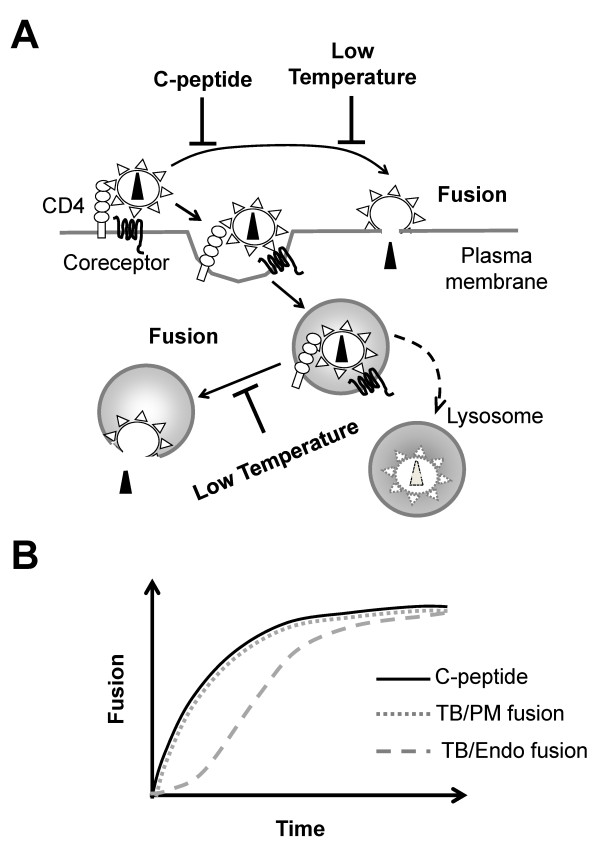
**Principal HIV-1 entry pathways and a functional strategy to determine the entry site**. (A) HIV-1 binds to CD4 along with a coreceptor and either undergoes fusion with the plasma membrane (PM) or with an endosomal membrane (Endo). While a C-peptide only inhibits fusion of non-internalized particles that have not fused at the time of its addition, low temperature (referred to as a temperature block, TB) is able to inhibit both PM and Endo fusion. (B) The time course of HIV-1 escape from fusion inhibitors during entry into and fusion with target cells. The virus sensitivity to C-peptide and TB would change at the similar rate, if fusion occurs at the plasma membrane (PM). By contrast, viruses entering through the endosomal route would acquire resistance to C-peptide but remain sensitive to the TB until they undergo fusion with an endosomal membrane.

We have previously designed a strategy to deduce the sites of entry for enveloped viruses that fuse in a low pH-independent manner [23]. The progression of HIV-1 entry through endocytosis and fusion was analyzed by adding either a membrane-impermeable inhibitory C-peptide, C52L [27], or by lowering the temperature. The HIV-1 gp41-derived C52L binds to the complementary gp41 domain formed/exposed following the Env binding to CD4 and coreceptors, thereby preventing the formation of the final 6-helix bundle structure [28]. Once gp41 completes folding into a stable 6-helix bundle, which occurs immediately after, but not before the fusion pore formation [29], fusion is no longer sensitive to C-peptide inhibition [29-31]. Therefore, C52L targets only surface-accessible viruses that have engaged CD4 or both CD4 and coreceptor, but have not progressed through the fusion step (Figure 1A). Note that binding of C-peptides to intermediate conformations of gp41 formed at the cell surface will block subsequent HIV-1 fusion with endosomal compartments. On the other hand, both cell-surface and endosomal fusion should be inhibited by low temperature (Figure 1A).

The above approach is based on the notion that endosomal fusion is usually delayed relative to the virus uptake. Thus, by comparing the rate of virus escape from a membrane-impermeable fusion inhibitor targeting cell surface-bound viruses to the rate of escape from low temperature that blocks all fusion events (referred to as a temperature block, TB), one can deduce whether the virus fuses at the plasma membrane or after entering by endocytosis [23]. Internalized viruses are protected from the peptide inhibitor, but not from the TB until the fusion step is completed (Figure 1A). Therefore, identical rates of virus escape from a peptide inhibitor and from TB (Figure 1B, solid vs. dotted line) would be consistent with direct fusion at the plasma membrane (PM) or with endosomal fusion immediately following the virus uptake. By comparison, delayed escape from the TB (Figure 1B, dashed line) implies that fusion occurs after a considerable lag time following the virus uptake and therefore productive entry must occur from endosomal compartments. In this case, protection from a peptide inhibitor likely reflects productive virus endocytosis and not the formation of 6-helix bundles as a result of fusion with the plasma membrane.

### HIV-1 exhibits strong preference for endosomal entry into engineered and natural target cells

Using the above functional strategy, we have previously found that HIV-1 enters target cells *via *endocytosis and fusion with endosomes [23]. Here, to test whether endocytic entry of HIV-1 was cell type-dependent, we analyzed the kinetics of HIV-1 fusion with several engineered and natural target cells. Virus-cell fusion was examined using the β-lactamase (BlaM) assay, which measures the transfer of viral core-associated enzyme into the cytosol [32]. HIV-1 cores pseudotyped with different HIV-1 envelope glycoproteins (Env) were bound to adherent or suspended target cells in the cold, and their fusion was initiated by shifting to 37°C. Fusion was stopped after varied times of incubation either by adding a fully inhibitory concentration of C52L or by chilling the samples (TB).

**Figure 2:**
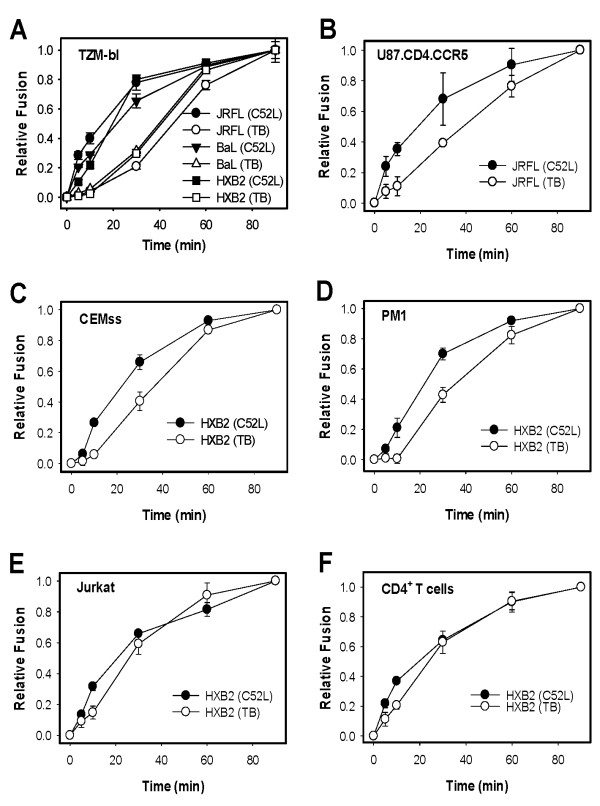
**Kinetics of HIV-1 fusion in engineered and natural target cells**. HXB2 pseudoviruses were spinoculated at 4°C onto TZM-bl (A), U87.CD4.CCR5 (B), CEMss (C), PM1 (D), Jurkat (E), and primary CD4^+ ^T cells (F). Particles pseudotyped with JRFL Env were also used in panels A and B, and BaL pseudotypes were employed in panel A. Fusion was allowed to proceed for 90 min at 37°C in DMEM/10% FBS and measured by the BlaM assay. Virus fusion was stopped either by adding C52L (1 μM) or by placing cells on ice (TB) after varied times of incubation. At the end of the incubation period, cells were placed on ice, loaded with the BlaM substrate, and the extent of fusion was measured after an overnight incubation at 12°C. Unless indicated otherwise, data points are means ± standard errors of the mean (SEM) from triplicate measurements.

We have previously demonstrated that HXB2 (CXCR4-tropic) and JRFL (CCR5-tropic) Env-pseudotyped particles enter TZM-bl indicator cells through an endocytic pathway, as evidenced by the delayed escape from TB compared to C52L [23]. Here we asked whether the same kinetic pattern can be observed for another CCR5-tropic HIV-1 isolate, BaL (Figure 2A). BaL-pseudotyped particles acquired resistance to C52L much earlier than to TB, implying that this Env also mediates the virus uptake and fusion with intracellular compartments. Interestingly, the respective kinetics of HXB2 and JRFL pseudovirus escape from the above inhibitors were close to those observed for BaL pseudotypes (Figure 2A). These results strengthen the argument that the tropism or the strain-specific features of HIV-1 Env does not determine the virus entry pathway in HeLa-derived target cells.

Next, we examined the sequence of steps leading to HIV-1 fusion in the U87.CD4.CCR5 cells expressing both CD4 and CCR5 [33]. Using single virus imaging, our group has previously shown that these cells, but not HeLa- or HOS-derived target cells, supported limited content release form HIV-1 pseudoviruses immobilized on coverslips [26]. Since in these experiments, target cells were detached from the culture plates and overlaid on immobilized viruses, we wanted to test whether HIV-1 pre-bound to adherent target cells would be able to release its content at the plasma membrane. Kinetic experiments revealed that escape of cell-bound JRFL viruses from the TB was markedly delayed compared to the acquisition of resistance to C52L (Figure 2B), suggesting that this virus entered the U87-derived cells primarily *via *an endocytic pathway.

We then asked whether the kinetics of HIV-1 entry and fusion with lymphoid cells would differ from those observed in engineered cell lines. In agreement with our previous results [23], HIV-1 entry into CEMss cells exhibited delayed resistance to TB relative to C52L (Figure 2C). A similar pattern was observed for PM1 cells (Figure 2D), whereas in Jurkat cells HIV-1 escape from TB was marginally delayed compared to the acquisition of resistance to the inhibitory peptide (Figure 2E). Finally, we compared the rates of HIV-1 escape from C52L and TB upon entry into primary CD4^+ ^T cells. As can be seen from 2F, there was modest but significant lag between the acquisition of resistance to the inhibitory peptide and to low temperature. This result suggests that at least a fraction of fusion-competent viruses escaped from C52L by undergoing endocytosis and fusing with intracellular compartments.

Collectively, our findings imply that HIV-1 enters epithelial, glial and lymphoid cells through an endocytic route. The variable lag between the virus uptake and fusion observed for these cells highlights the role for cell type-dependent endosomal trafficking in delivering HIV-1 into fusion-permissive compartments.

### Single HIV-1 tracking reveals endosomal entry and fusion with lymphoid cells

To verify HIV-1 entry through an endocytic pathway, as suggested by the BlaM assay, we imaged the fusion of single pseudoviruses with lymphoid cells. Co-labeling pseudoparticles with a small releasable viral content marker tagged with GFP and with the lipophilic dye DiD incorporated into the viral envelope allowed for pinpointing the sites of virus entry [23]. This approach takes advantage of the tremendous difference in the surface area of the plasma membrane and an endosome, so that virus hemifusion/fusion would lead to infinite dilutions (disappearance) of the lipid marker at the plasma membrane vs. limited dilution (retention) in endosomes. The viral content release marks the pore formation that results in disappearance of punctate signal through dilution within the cytosol, irrespective of the fusion site. We have previously shown that cell surface-bound HIV-1 particles exchange lipids with the plasma membrane, but do not release their content, an outcome suggestive of a hemifusion phenotype [23,26,34]. Internalized virions, on the other hand, underwent full fusion with endosomes. This experimental approach has been validated using viruses known to enter cells *via *endocytosis [23].

**Figure 3:**
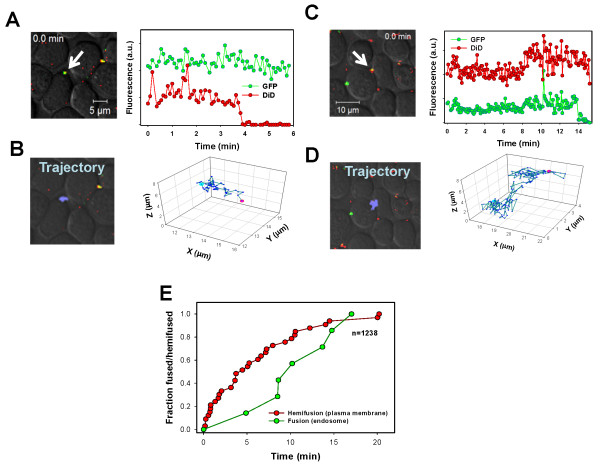
**Imaging the HIV-1 fusion with lymphoid cells**. HIV-1 JRFL Env pseudotyped MLV particles co-labeled with Gag-GFP and DiD were bound to CEM.NKR-CCR5.Luc cells by spinoculation at 4°C. Virus uptake was triggered by raising the temperature to 37°C just prior to imaging. (A) Hemifusion events at the cell surface were characterized by the loss of the DiD signal while the GFP-tagged viral content was retained. (B) Hemifusion with the plasma membrane is associated with restricted particle motility. (C) Upon endosomal fusion, the membrane marker, DiD, remained within the recipient endosome while the Gag-GFP signal was rapidly lost. (D) The virus shown in (C) underwent more extensive movement. (E) Kinetics of the individual hemifusion (red line) or fusion (green line) events plotted as cumulative distributions as a function of time. Arrows in A and C indicate the tracked co-labeled virus.

To further confirm that HIV-1 enters lymphoid cells through an endocytic pathway, we tracked entry of single JFRL Env pseudoviruses into CEM.NKR-CCR5.Luc cells stably expressing CCR5. Virus uptake and fusion were triggered by quickly raising the temperature in the image field to 37°C (Figure 3A and 3C, time = 0 sec), as described in [23]. Due to the limited cytoplasm around the nucleus of CEM-derived cells, it was virtually impossible to visualize the virus trafficking from the cell periphery toward the nucleus based on spatial displacement. Importantly, however, we were able to deduce the site of HIV-1 entry into these cells by monitoring the extents of dilution of viral lipid and content markers ([23] and see below).

Similar to TZM-bl cells [23], two types of viral marker redistribution events were observed with CEM-derived cells. First, the membrane marker signal (red) quickly dropped to the background level while the signal from the GFP-tagged viral content (green) remained relatively constant (Figure 3A). The lack of viral content release concomitant with a drop of the lipid marker is suggestive of virus hemifusion at the cell surface. Particles undergoing hemifusion usually exhibited limited movement before and immediately after the lipid mixing step (Figure 3B). In fact, the limited displacement of these viral particles from their initial position was often caused by cell motility associated with a steep increase in temperature at the beginning of the experiment. The second type of events was characterized by disappearance of the viral content marker while the fluorescence of the membrane dye remained virtually unchanged indicating endosomal fusion (Figure 3C). In this case, we ordinarily observed more extensive virus movement before the content release events were detected (Figure 3D).

Out of the total 1238 co-labeled viral particles, 29 (2.3%) appeared to hemifuse with the plasma membrane, while only 7 (0.6%) underwent full fusion within endosomes (Figure 3E). Thus, the efficiency of JRFL fusion with the CEM-derived cell line was relatively low. As with TZM-bl cells, JRFL fusion was primarily initiated at the cell surface, resulting in lipid mixing but not the viral content release. Also, as previously observed [23], the kinetics of lipid mixing at the plasma membrane was considerably faster than the viral content release from endosomes (Figure 3E), consistent with the delayed endocytic entry of this virus. Importantly, the faster rate of hemifusion at the surface of TZM-bl and CEM.NKR-CCR5.Luc cells compared to that of the content release shows that the lack of HIV-1 fusion with the plasma membrane was not due to a quick particle internalization, as suggested in [24]. Clearly, HIV-1 Env often initiates fusion with the plasma membrane earlier than the virus gets internalized but fails to create a functional fusion pore. In summary, both the BlaM and single virus imaging assays (Figures 2 and 3) imply that HIV-1 enters into a number of cell lines and into primary CD4^+ ^T cells through endocytosis.

### Synchronizing HIV-1 entry through trapping the virus-CD4-coreceptor complexes at the cell surface does not favor fusion with the plasma membrane

We sought to optimize and accelerate the HIV-1 fusion reaction in an attempt to redirect it from an endocytic pathway to the plasma membrane. This was achieved by pre-incubating the viruses with target cells at sub-threshold temperature for fusion (18-24°C), which resulted in creation of a temperature-arrested stage (TAS) [23,30]. Under these conditions, a large fraction of Env engages a requisite number of CD4 and coreceptors, as evidenced by resistance of fusion to high concentrations of CD4 [35] and coreceptor binding inhibitors added at TAS [23,30]. Our previous studies using a cell-cell model demonstrated that fusion from TAS induced by raising the temperature was markedly accelerated compared to control cells pre-incubated at 4°C [30,36]. We, therefore, reasoned that HIV-cell fusion from this intermediate stage would also be accelerated, thus favoring direct fusion with the plasma membrane prior to the virus uptake.

**Figure 4:**
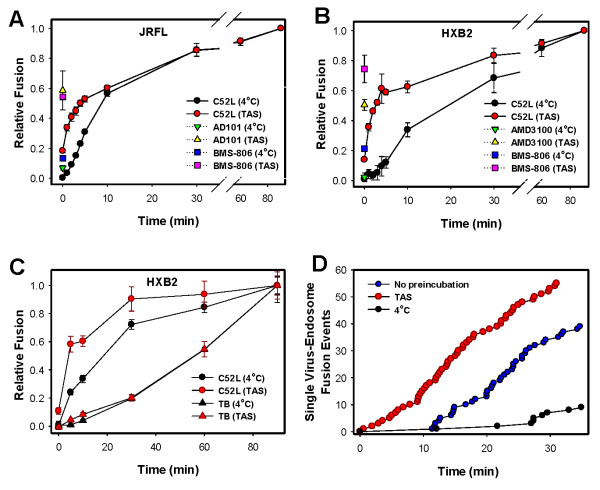
**Synchronizing the HIV-1 fusion by pre-forming Env-CD4-coreceptor complexes does not redirect fusion to the plasma membrane**. JRFL (A) or HXB2 (B) pseudoviruses carrying BlaM-Vpr were pre-bound to TZM-bl cells in the cold and further incubated for 2.5 h at 20.5°C (JRFL), 22-24°C (HXB2) to create a temperature-arrested stage (TAS) or at 4°C (control). Fusion was induced by raising the temperature to 37°C in DMEM/10% FBS. The extent of the functional CD4 and coreceptor engagement by the virus was evaluated by adding the respective fusion inhibitors BMS-806 (10 μM, CD4 binding), AD101 (10 μM, CCR5 binding) or AMD3100 (10 μM, CXCR4 binding) and measuring the BlaM activity. Alternatively, the time course of HIV-1 escape from C52L (1 μM) was measured by adding the peptide at indicated time points. (C) Similar to panel B, but, in addition to the time course of HXB2 escape from C52L, the kinetics of acquisition of resistance to the TB was also determined. (D) HXB2 pseudotyped viruses labeled with the MLV Gag-GFP and DiD were bound to TZM-bl cells in the cold and either immediately used for imaging experiments (blue circles) or further incubated for 2.5 h either at 4°C or, to create TAS, at 24°C. Fusion was trigged by raising the temperature to 37°C at the onset of imaging. Single viruses were tracked and individual fusion events quantified as described in Materials and Methods. The differences in the kinetics of the virus escape from C52L in panels B and C reflect the experimental variability due to differences in the viral stock and the cell passage numbers.

Pseudoviruses bearing JRFL or HXB2 Env were pre-bound to TZM-bl cells at 4°C, and TAS was created by incubation at a temperature just below the threshold for fusion for 2.5 h. The empirically determined optimal temperatures for creating TAS were ~21°C for JRFL and ~24°C for HXB2. Under these conditions, only 10-20% of fusion-competent JRFL or HXB2 particles acquired resistance to C52L (Figure 4A and 4B, red circles), apparently due to the limited virus uptake/fusion during the extended pre-incubation at sub-threshold temperature. At the same time, 50-75% of virions became resistant to CD4 and coreceptor binding inhibitors added immediately before raising the temperature from TAS (pink vs. yellow symbols, respectively), confirming that a large portion of viruses had engaged both molecules. Control samples kept for 2.5 h at 4°C exhibited minimal or no resistance to HIV-1 entry inhibitors, consistent with the lack of Env-CD4-coreceptor interactions or virus uptake (black, blue and green symbols). We next tested if the creation of TAS accelerated HIV-1 entry/fusion, as was the case for the cell-cell fusion process [30,36]. When fusion was initiated by shifting to 37°C, viruses captured at TAS acquired resistance to C52L faster than control viruses pre-incubated at 4°C (Figure 4A and 4B). This effect was more pronounced for HXB2 compared to JRFL pseudoviruses. However, as we have previously shown [23], escape from C52L alone does not unambiguously prove that fusion occurs at the cell surface. If fusion takes place in endosomes, the virus resistance to C52L would be acquired at an earlier time through productive endocytosis (Figure 1).

In order to assess the HIV-1 entry pathway after creating TAS, we performed comparative measurements of the escape kinetics from C52L and TB. These measurements were carried out using HXB2 particles, since HXB2 exhibited a more pronounced acceleration of entry from TAS (Figure 4A and 4B). As in Figure 4B, after creating TAS, resistance to C52L was acquired faster than in control samples (Figure 4C, red vs. black circles). However, the rates of virus escape from TB were markedly delayed relative to escape from C52L, both for TAS-arrested and control samples (triangles vs. circles). We thus concluded that, in spite of engaging CD4 and coreceptors at the cell surface, the virus still failed to fuse with the plasma membrane. Instead, the creation of TAS appeared to accelerate productive HIV-1 endocytosis, whereas fusion with endosomes (escape from TB) occurred at the same rate as in control experiments (red vs. black triangles). In other words, the rate of endosomal fusion was virtually independent of the rate of virus uptake.

In parallel experiments, we imaged single HXB2 pseudovirus fusion with TZM-bl cells after creating TAS and in control samples pre-incubated in the cold for 2.5 hours. However, very few fusion events (content release from endosomes) were detected for cold pre-incubated cells following the subsequent incubation at 37°C for 35 minutes, whereas fusion was consistently observed after creating TAS (Figure 4D, black vs. red circles). The shorter incubation time at 37°C and the necessity to keep cells on ice prior to imaging (see Materials and Methods) could contribute to the lower tolerance of single virus fusion to pre-incubation in the cold. To aid the assessment of the effect of TAS on the kinetics of HIV-1 fusion, we compared cells pre-incubated at 24°C to those imaged immediately after the virus binding step (Figure 4D, blue circles). This comparison revealed that, in agreement with the BlaM results, the rate of single virus fusion after TAS (proportional to the slope of plots in Figure 4D) was virtually identical to that without pre-incubation (Figure 4D, red vs. blue circles). However, in control samples fusion commenced after about a 10 min lag, whereas fusion after TAS proceeded without a delay. The nearly identical kinetics of fusion measured by the BlaM assay with or without creating TAS (Figure 4C, triangles) indicates that the BlaM activity might occur after an additional rate-limiting step, such as a slow dilation of fusion pores between HIV-1 and endosomes. Importantly, the creation of TAS neither eliminated the kinetic differential between C52L and TB in BlaM experiments nor allowed single virus fusion at the cell surface in imaging experiments.

Collectively, our data imply that synchronization of HIV-1 fusion through formation of Env-CD4-coreceptor complexes at the cell surface does not favor direct fusion at that site. These conditions accelerate productive endocytosis (escape from C52L), while the rate of fusion with endosomes (escape from TB) remains unaltered. The observation that after creation of TAS HIV-1 appears to spend more time within intracellular compartments prior to undergoing fusion highlights the role of endosome trafficking/maturation in facilitating the virus entry.

### Blocking HIV-1 uptake by a dynamin inhibitor allows lipid mixing but not full fusion with the plasma membrane

Another strategy to redirect HIV-1 fusion to the plasma membrane was based on preventing the virus endocytosis at physiological temperature. We used the small-molecule dynamin inhibitor, dynasore [22], which suppresses HIV-1 endocytosis and fusion [23]. Virions trapped at the cell surface in the presence of this inhibitor quickly lost their fusion competence, as evidenced by the lack of recovery of the BlaM signal following the removal of this inhibitor (supplemental material to [23]). However, whether or not HIV-1 underwent hemifusion (lipid mixing) under these conditions remained unclear, owing to the strong quenching of the viral DiD fluorescence by dynasore ([23], data not shown).

**Figure 5:**
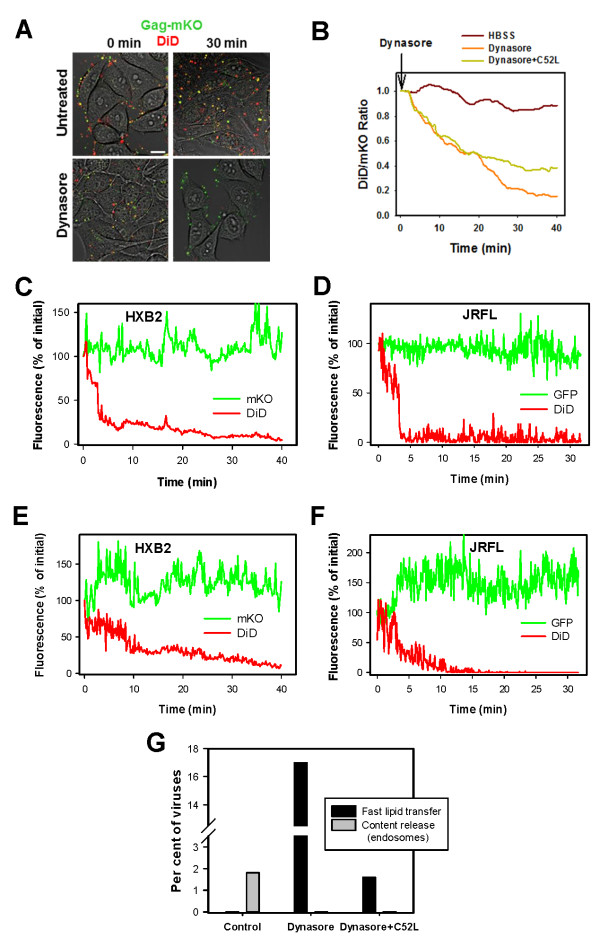
**HIV-1 hemifuson with the plasma membrane can be distinguished from dynasore-mediated quenching of the viral membrane marker**. (A) TZM-bl cells were pretreated with 60 μM dynasore (Santa Cruz) in HBSS^++ ^and centrifuged with HXB2 Gag-mKO/DiD virus. Cells were washed with HBSS^++ ^and imaged in the same buffer (upper panel), in 60 μM dynasore (lower panel), or in 60 μM dynasore + 1 μM of C52L (not shown) for 30 min at 37°C. mKO is pseudocolored green and DiD is pseudocolored red. Scale bar is 20 μm. (B) The changes in DiD intensity induced by dynasore were evaluated based on the mean fluorescence of at least 100 virions positive for both Gag-mKO and DiD. To compensate for the focal drift and imaging artifacts related to the cell movement, the ratio of mean DiD/mKO fluorescence signals is plotted over time. MLV Gag-mKO/DiD or Gag-GFP/DiD particles pseudotyped with HXB2 Env (C and E) or JRFL Env (D and F) were bound to TZM-bl cells pretreated with 60 μM dynasore in HBSS^++^. Individual viruses were tracked and analyzed for changes in fluorescence intensity. Single particle tracking in the presence of dynasore demonstrated either the fast DiD decay (C andD) or slow decay (E and F) phenotypes in the presence of dynasore. (G) The fraction of co-labeled HXB2 pseudoviruses undergoing fast lipid transfer or endosomal fusion (content release) in control experiments or in the presence of 60 μM dynasore with or without 1 μM C52L.

We further examined the nature of the dynasore-imposed block of HIV-1 fusion. In agreement with the previous results [23], dynasore strongly quenched the DiD signal (Figure 5A), as well as the fluorescence of its green analogue, DiO, and of the chemically distinct membrane probe, R18 (Additional File 1: Figure S1A-D), showing that the quenching effect was not specific for the chemical structure or spectral characteristics of DiD. In contrast to lipophilic dyes, dynasore did not alter the signal from the viral content markers, Gag-mKO or Gag-GFP (Additional File 1: Figure S1A-B). However, other dynamin inhibitors, dynole 34-2 and MiTMAB, did not affect the DiD fluorescence (Additional File 1: Figure S1E-G). Analysis of the mean DiD fluorescence of cell-bound viruses confirmed the loss of the red signal, while the mKO fluorescence was not significantly affected by dynasore (Figure 5B). Importantly, we did not detect any viral content release events at the cell surface with or without dynasore (Figure 5).

In order to determine whether, in addition to the direct DiD quenching by dynasore, a fraction of this signal could be lost through HXB2-cell hemifusion, imaging experiments were performed in the presence of both dynasore and a fully inhibitory concentration of C52L. As expected, the overall fluorescence decayed when fusion was blocked by C52L (Figure 5B), but the diminution of the lipid dye fluorescence was less pronounced than in the presence of dynasore alone. When C52L was added during imaging, the DiD/mKO ratio dropped by ~60% within the 40 min-incubation period, as compared to ~85% in the absence of the peptide inhibitor. The sensitivity of DiD quenching to C52L indicates that a sizeable fraction of events resulting in disappearance of DiD puncta (perhaps as much as 25%, Figure 5B) are due to the HIV-1 Env-mediated release of the lipid marker into the plasma membrane. This is in contrast to rare lipid mixing events observed in the absence of dynamin inhibitor [23].

To verify that HIV-1 can exchange lipids with the plasma membrane in the presence of dynasore, we tracked single HXB2 and JRFL particles. This analysis revealed two distinct types of DiD disappearance events - fast, exhibiting a nearly instantaneous drop of fluorescence (Figure 5C, D), and slow, lasting 10 min or longer (Figure 5E, F). When experiments were performed in the presence of both dynasore and C52L, fast events were virtually abrogated while the slow events remained. Figure 5G shows the inhibition of the fast DiD decay events in the presence of dynasore and C52L and concomitant disappearance of already infrequent content release from endosomes. In contrast, slow DiD decay was not noticeably affected by the presence of C52L (data not shown). These findings are consistent with the notion that the slow loss of DiD fluorescence is caused by dynasore, whereas the fast DiD decay reflects transfer of viral lipids into the plasma membrane mediated by Env. By definition, lipid mixing in the absence of content release corresponds to a hemifusion phenotype.

We found that 17% of single viruses quickly released DiD into the plasma membrane (Figure 5G), a number that was comparable to the estimated fraction of hemifusion events in the presence of C52L based on the global image analysis (Figure 5B). In summary, through trapping HIV-1 at the cell surface, we increased the occurrence of Env-mediated lipid mixing. However, in spite of the exaggerated hemifusion phenotype in the presence of dynasore, viruses failed to undergo full fusion with the plasma membrane.

### Dynasore incorporates into lipid membranes without significantly affecting their propensity to fuse

**Figure 6:**
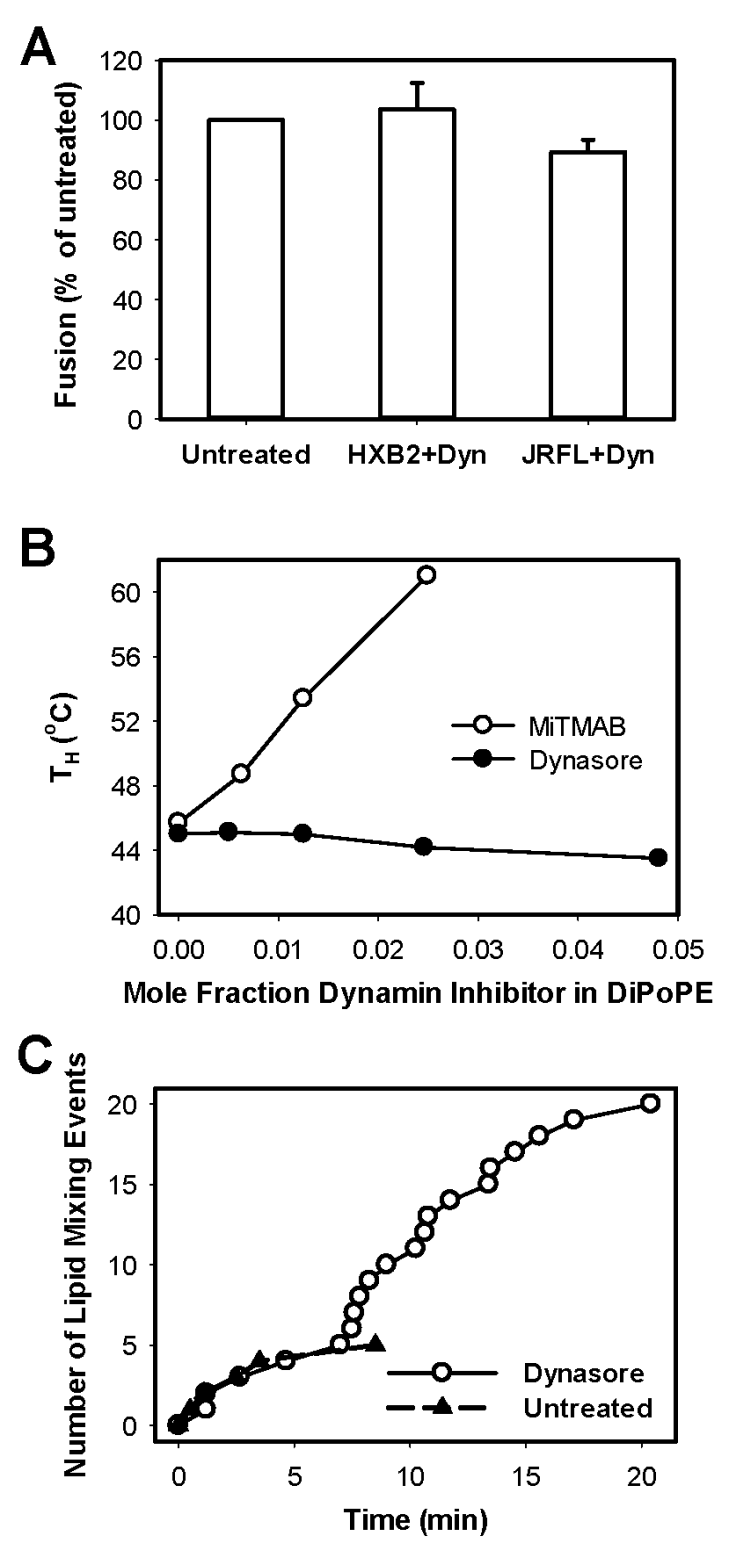
**The effect of dynasore on the HIV-1 fusion-competence and on the curvature of lipid bilayers is minimal**. (A) Concentrated stocks of JRFL and HXB2 pseudoviruses (1·10^7 ^IU/ml) were pretreated with 80 μM dynasore in DMEM for 20 min at 37°C. Virus inoculum was diluted 20-fold prior to adding to TZM-bl cells and centrifuging at 4°C to aid binding. Cells were washed, and fusion was triggered by shifting to 37°C for 90 min in DMEM/10% FBS and measured by the BlaM assay. (B) Dynasore and MiTMAB were mixed with DiPoPE lipid and the temperature of lamellar-H_II _transition (T_H_) was measured by differential scanning calorimetry. Each sample contained 5 mg of DiPoPE and varying amounts of dynasore or MiTMAB suspended in 0.8 ml PIPES buffer (20 mM PIPES, 1 mM EDTA, 140 mM NaCl, pH 7.4). The sample and the same reference buffer were added to separate cells. Temperature scans were made from 15 to 55°C at a 1°/min scan rate. The transition temperature for each mixture was determined using a curve fitting program DA-2 supplied by Microcal, Inc. (Northampton, MA). This transition temperature (T_H_) is plotted against the mole fraction of drug added. (C) The waiting time from triggering fusion to HXB2 hemifusion events (fast DiD decay) with TZM-bl cells was determined in the presence of dynasore and plotted as the number of events over time (circles). For comparison, the kinetics of hemifusion with the plasma membrane in the absence of dynasore is re-plotted from [23] (triangles).

The DiD-quenching activity of dynasore prompted us to investigate its effect on the stability of lipid bilayers and their propensity to undergo hemifusion/fusion. We first tested whether dynasore could directly inactivate the virus. Pre-treatment of viruses with dynasore did not significantly decrease virus-cell binding [23] or the fusion activity (Figure 6A) compared to control viruses. We also did not detect adverse effects of dynasore on infectivity of immobilized viruses (data not shown). Because dynasore did not exhibit a direct virucidal effect on HIV-1, we concluded that the previously observed virus inactivation by this compound [23] was dependent on HIV-cell contact.

We then asked whether, in addition to quenching the fluorescence of lipid dyes, dynasore can also affect the propensity of virus and/or cell membranes to undergo hemifusion/fusion. Toward this goal we measured the temperature of the lamellar-inverted hexagonal (H_II_) phase transition (T_H_) in pure lipid bilayers in the presence of dynasore and another dynamin inhibitor, MiTMAB. Compounds that lower the T_H _promote negative membrane curvature required for the formation of a stalk intermediate between two fusing bilayers, whereas compounds that raise the transition temperature are inhibitory for fusion [37-40]. To determine how modulators of dynamin activity affect membrane curvature, we studied the ability to shift the T-_H _of a model lipid, dipalmitoloeoyl phosphatidylethanolamine (DiPoPE). This lipid has a T_H _at about 45°C and is convenient for these types of studies [41-43]. When dynasore was mixed with DiPOPE, it lowered the T_H _of this lipid in a dose-dependent manner (Figure 6B). However, this effect was weak compared to known promoters of negative curvature, such as diacylglycerol [44]. In contrast, MiTMAB, which inhibits dynamin activity by preventing its binding to phospholipids [45], strongly increased the T_H _(Figure 6B), in agreement with the previous study [46]. Thus, MiTMAB promotes strong positive curvature and is thereby expected to inhibit bilayer hemifusion, whereas dynasore confers weak negative curvature that is unlikely to be of consequence for HIV-1 fusion.

To test whether the dynasore's modest effect on lipid bilayers can modulate HIV-1 Env-mediated fusion, we analyzed the kinetics of virus-cell hemifusion in the presence of this compound. The waiting times from initiating the virus fusion by raising the temperature to each fast lipid mixing event were measured and plotted as the number lipid mixing events over time. We found that lipid mixing between HIV-1 and the plasma membrane occurred at the same rate, irrespective of the presence of dynasore (Figure 6C). Consistent with our previous study [23], HXB2 pseudoviruses rarely exchanged lipids with the plasma membrane. The number of observed hemifusion events in the absence of dynasore appears truncated, likely due to the virus clearance from the cell surface through endocytosis. The invariant kinetics of hemifusion suggests that dynasore itself does not facilitate membrane merger, in agreement with its weak effect on the lipid curvature (Figure 6B). We therefore concluded that the increased number of hemifusion events in the presence of dynasore was caused by trapping of viruses at the cell surface and thus extending the window of opportunity for lipid mixing. Notably, the previously observed loss of HIV-1 fusion activity following a pre-incubation of cell-bound pseudoviruses with dynasore [23] occurred over the same time course (20 min at 37°C) as the hemifusion events (Figure 6C). These data suggest that dead-end hemifusion is responsible for inactivation of cell-bound HIV-1 in the presence of dynasore.

### Dynasore more potently blocks HIV-1 endocytosis than subsequent fusion with endosomes

**Figure 7:**
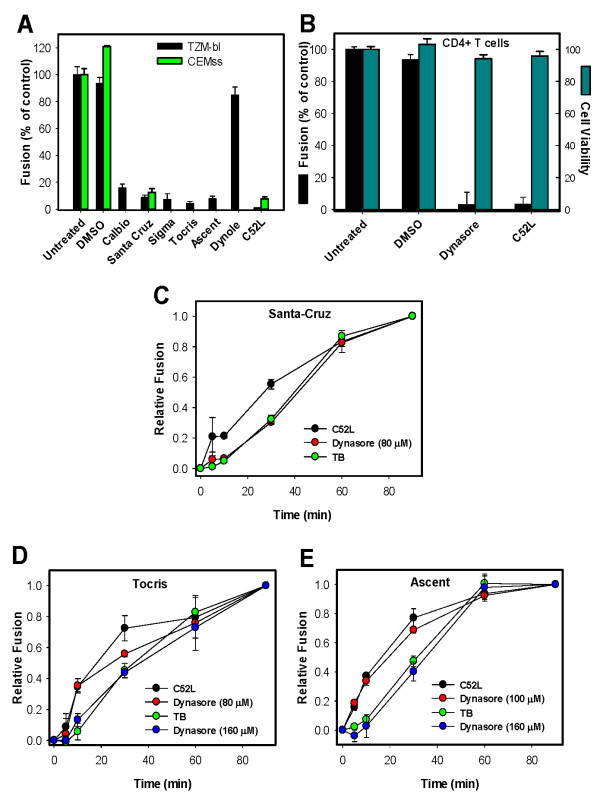
**Dynamin inhibitors from different manufacturers have similar effects on HIV-1 uptake and fusion with endosomes**. HXB2 pseudoviruses were bound to TZM-bl or CEMss cells at 4°C (see Methods), and fusion was initiated by shifting to 37°C for 90 min (in DMEM) and measured by the BlaM assay. (A) Fusion with TZM-bl cells (black bars) in the presence or absence of 80 μM dynasore or dynole 34-2 in DMEM. Dynasore from Ascent was used at 100 μM. The effects of DMSO (0.1% v/v) and 80 μM dynasore (Santa Cruz) on fusion with CEMss cells are also shown (open bars). The extent of fusion in the presence of 1 μM C52L is shown for comparison. (B) Inhibition of HXB2 pseudovirus fusion with primary CD4^+ ^T cells by 80 μM dynasore (Santa Cruz) and 1 μM C52L (filled bars). The effect of DMSO, dynasore and C52L on cell viability, as determined by the MTS assay, is shown by open bars. (C-E) The kinetics of HXB2 escape from C52L, dynasore and the temperature block (TB) applied at indicated times of incubation at 37°C. The background fusion (~15% of the maximal signal) in the presence of dynasore was subtracted from the dynasore kinetics data to ease the comparison with the C52L and TB kinetics. Data points are means and SEM of combined triplicate measurements from three independent experiments.

If an inhibitor can target more than one step of virus entry, time of addition experiments would reveal the latest step of this process affected by inhibitor. We have previously reported that HIV-1 escapes from dynasore and the TB with nearly identical kinetics, while acquiring resistance to a peptide inhibitor at an earlier time [23]. This finding thus implies that dynasore not only blocks HIV-1 endocytosis, but also inhibits the virus-endosome fusion step. However, a fluorescence quenching activity of dynasore raised concerns regarding possible off-target effects of this compound. We therefore re-tested the effect of dynasore from different commercial sources on HIV-1 fusion. At 80 μM, all tested preparations efficiently blocked transferrin uptake in TZM-bl cells without exhibiting significant cytotoxicity (Additional File 2: Figure S2A-G and Additional File 3: Figure S3A). At this concentration, dynasore preparations effectively inhibited fusion between HXB2 pseudoviruses and TZM-bl cells (Figure 7A). By contrast, another dynamin inhibitor dynole 34-2, when used at 80 μM, only partially inhibited transferrin uptake (Additional File 2: Figure S2H, I) and exhibited only a minor effect on HIV-1 fusion (Figure 7A) or endocytosis (Additional File 4: Figure S4). Since higher concentrations of dynole 34-2 were toxic to TZM-bl cells (Additional File 3: Figure S3B) we did not further investigate this compound. The effect of dynasore on HXB2 fusion with CEMss cells was also evaluated.

We found that dynasore inhibited HIV-1 fusion with CEMss cells as effectively as with TZM-bl cells (Figure 7A, green bars) without significantly affecting the cell viability (data not shown). Furthermore, 80 μM of dynasore abrogated HXB2 fusion with primary CD4^+ ^T cells (Figure 7B, filled bars). The viability of these cells was not compromise by this concentration of dynamin inhibitor (Figure 7B, dark cyan bars). The potent block of HIV-1 fusion with primary T cells by dynasore further supports the endocytic entry route of this virus.

A considerable lag between HIV-1 escape from C52L and the TB in TZM-bl cells (Figure 2) indicates that virus might rely on endosomal trafficking and maturation in order to undergo fusion. To test whether dynasore could exert its inhibitory effect on HIV-1 fusion by interfering with cellular trafficking, we imaged single virus movement within target cells and applied a fully inhibitory dose of dynasore at an arbitrary time point. The lack of noticeable effect on virus trafficking (Additional File 5: movie 1) argued against the possibility that dynasore could interfere with molecular motors or cytoskeletal components involved in virus trafficking. Using cells transfected with GFP-Rab5, we also found that this compound did not affect a general movement pattern of endosomes (Additional File 6: movie S2). To conclude, we did not detect any obvious off-target effects of dynasore in TZM-bl cells.

To evaluate the last step of HIV-1 entry targeted by dynasore, we compared the rates of virus escape from C52L (receptor-mediated endocytosis), TB (fusion with endosomes) and from dynasore added at varied times of virus-cell incubation at 37°C. Out of five dynasore preparations tested, only the Santa Cruz compound appeared to inhibit HIV-endosome fusion step (coinciding with the TB-sensitive step) at 80 μM, similar to dynasore previously obtained from T. Kirchhausen (Figure 7C and [23]). For other tested dynasore preparations, the rate of HIV-1 escape from this inhibitor tended to be closer to that for C52L (Figure 7D, E and data not shown) than to the TB, consistent with inhibition of receptor-mediated virus endocytosis. However, when the concentration of different dynasore preparations was increased to 160 μM, delayed escape from this compound was reproducibly observed (Figure 7D, E), suggesting that the higher dose of dynasore targeted the HIV-endosome fusion step.

Taken together, our results showed that dynasore reproducibly blocked HIV-1 endocytosis and fusion, but that the rate of virus escape from this compound varied somewhat depending on the dynasore preparation. The remarkable switch between inhibiting receptor-mediated endocytosis to blocking virus-endosome fusion that occurred in a narrow range of dynasore concentrations could be indicative of a more stringent requirement for dynamin to pinch off endocytic vesicles compared to its apparent role in aiding the HIV-1 fusion with endosomes. Interestingly, dynasore has been shown to block transferrin uptake in HeLa cells at lower doses compared to those required to inhibit HIV-1 endocytosis ([22] and Fig. 8), perhaps suggesting the involvement of factors other than dynamin in virus internalization.

## Discussion

A large number of viruses enter host cells through intracellular compartments where low pH and/or cellular proteases activate the viral fusion proteins, permitting the release of the virus' genome from intracellular compartments [23,47,48]. Studies of virus entry helped identify new routes of endosomal trafficking. However, the entry pathways of enveloped viruses that do not rely on low pH to activate their fusion proteins are less well understood. These viruses can enter both *via *direct fusion with the plasma membrane and endocytosis. Indeed, several viruses undergoing receptor-mediated fusion at neutral pH appear to rely on endocytosis for productive entry [49-51]. Studies aimed at determining the HIV-1 entry site yielded contradicting evidence for and against the involvement of endocytosis in productive entry of this virus [5-7,12,20,21]. The fact that HIV-1 does not rely on low pH to infect most target cells [8,18,19] has confounded the identification of its entry pathways. Non-invasive functional approaches developed by our group [23] showed that, surprisingly, HIV-1 fusion initiated at the cell surface was blocked at a post lipid mixing step, whereas internalized viruses underwent complete fusion with endosomes. However, the molecular basis for the strong preference for entry from intracellular compartments was not delineated and some evidence supporting the HIV-1 fusion with the plasma membrane, including the virus' ability to fuse adjacent cells [9], remained controversial.

In this study, we provided further evidence that HIV-1 enters distinct permissive cell lines and primary CD4^+ ^T cells through endocytosis and fusion with endosomes. This conclusion was supported by a lag between HIV-1 escape from C52L and from the TB, which was cell type-dependent, but independent of coreceptor tropism (Figure 2A). This lag was also not affected by attempts to synchronize fusion by creating TAS (Figure 4C). The cell type-dependent difference of the kinetics of virus escape from C52L and low temperature is consistent with the essential role of endosome trafficking and maturation for HIV-1 entry. The HIV's reliance on endocytic pathways is further supported by single virus imaging in lymphoid cells, in which we observed hemifusion with the plasma membrane and complete fusion with endosomal compartments. More importantly, attempts to redirect HIV-1 fusion to the plasma membrane, either by synchronizing the multistep fusion reaction or by trapping the virus on the cell surface using dynasore, were unsuccessful. In spite of the ability to quench the fluorescence of lipophilic dyes, dynasore does not strongly affect the properties of lipid bilayers that are essential for fusion or modulate the kinetics of hemifusion at the cell surface. It is thus unlikely that the lack of complete fusion between HIV-1 and the plasma membrane is due to an inadvertent effect of dynasore.

On the other hand, a short lag between HIV-1 escape from C52L compared to escape from the TB observed in Jurkat and in primary T cells is consistent both with endocytic entry and with fusion at the cell surface. However, based on the single virus imaging data (Figure 3), we propose that HIV-1 fuses with early endosomal compartments of lymphoid cells. The potent inhibition of HIV-1 fusion with CEMss cells by dynasore (Figure 7A) supports this notion. Future studies of single virus fusion in cells exhibiting minimal kinetic differential between the virus escape from C52L and TB in BlaM experiments should reveal whether these cells support HIV-1 fusion with the plasma membrane.

A recent study has demonstrated that the centrifugal force applied to cells during spinoculation with viruses enhances HIV-1 infection by promoting actin dynamics [52]. Although virus spinoculation was used in our studies, actin remodeling should only promote direct fusion at the cell surface [53]. It thus appears that HIV-1 fusion with the plasma membrane is disfavored compared to fusion with endosomal compartments. We have previously speculated that a handful of Env glycoproteins incorporated into a virion [54] may not be able to promote dilation of a fusion pore formed at the cell surface, whereas the viral content release from intracellular compartments is aided by yet unknown endosomal factor(s) [23]. Based on the late escape of HIV-1 from inhibition by dynasore, which coincided with the escape from the temperature block ([23] and Figure 7), it appears that dynamin can be involved in the virus-endosome fusion step. In other words, this GTPase can control HIV-1 entry at two check points - receptor-mediated uptake and fusion with endosomes. It remains to be determined whether dynasore can also inhibit HIV-endosome fusion in T cells.

The requirement for a somewhat higher concentration of dynasore to block HIV-endosome fusion compared to a dose that inhibits productive endocytosis (Figure 7D and 7E) indicates that the fusion step may be less reliant on dynamin activity than virus uptake. The possibility of the dynamin involvement in HIV-endosome fusion is not surprising, considering that this protein plays a role in a number of cellular processes besides vesicle budding from the plasma membrane (reviewed in [55]). In particular, dynamin has been shown to regulate receptor trafficking from late endosomes to lysosomes [56], as well as from late endosomes to the Golgi and ER [57-59]. Furthermore, dynasore has been reported to block SV40 infection by inhibiting endosomal trafficking downstream of a nocodazole-sensitive step [60]. It is thus possible that dynamin directly or indirectly promotes dilation of a fusion pore connecting the HIV-1 envelope and an endosomal membrane.

## Conclusions

This study expanded and reinforced our previous finding that HIV-1 enters susceptible cells by fusing with endosomes. First, we demonstrated the existence of a lag between HIV-1 uptake and fusion in cell lines of different origin and in primary CD4^+ ^T cells. Second, consistent with HIV's reliance on endosomal trafficking, the progression of fusion beyond the temperature-sensitive steps occurred at different rates in epithelial, glial and lymphoid cells. Third, the time of addition experiments using dynasore implied that this compound blocked fusion of internalized viral particles. Fourth, imaging single virus fusion with lymphoid cells identified endosomes as HIV-1 entry sites. Finally, in an attempt to redirect HIV-1 fusion to the plasma membrane, we blocked the virus uptake. HIV-1 particles trapped on the cell surface released a lipid marker but failed to undergo complete fusion. Collectively, our data strongly support the notion that HIV-1 infects cells by entering and fusing with endosomes and that dynamin plays a role in virus uptake and in the subsequent virus-endosome fusion.

## Methods

### Cells and Reagents

HeLa-derived indicator TZM-bl cells expressing CD4, CXCR4 and CCR5 (donated by Drs. J.C. Kappes and X. Wu [61]), human T4-lymphoblastoid CEMss cells (donated by Dr. P. Nara [62]), CEM-NKR-CCR5-Luc cells (donated by Drs. J. Moore and C. Spenlehauer [63]), PM-1 cells (donated by Drs. P. Lusso and R. Gallo [64]) and human astroglioma U87-MG-derived U87.CD4.CCR5 cells (donated by Dr. H. Deng and Dr. D.R. Littman [33]) were obtained from the NIH AIDS Research and Reference Reagent Program. Jurkat and human embryonic kidney 293T/17 cells were obtained from the ATCC (Manassas, VA). TZM-bl were grown in Dulbecco's Modified Eagle Medium (DMEM, Cellgro, Manassas, VA), whereas CEMss, CEM-NKR-CCR5-Luc, Jurkat, and PM-1 cells were grown in RPMI-1640 (Cellgro) supplemented with 10% Fetal Bovine Serum (FBS, HyClone Laboratories, Logan, UT) and 100 U penicillin-streptomycin (Gemini Bio-Products, West Sacramento, CA). U87.CD4.CCR5 were maintained in DMEM supplemented with 15% FBS, 100U penicillin-streptomycin, 0.5 mg/ml G418 sulfate (Cellgro), and 1 μg/ml puromycin (Sigma, St. Louis, MO). HEK 293T/17 cells were maintained in DMEM supplemented with 10% FBS, 100 U penicillin/streptomycin and 0.5 mg/ml G418 sulfate. Primary peripheral blood mononuclear cells (PBMCs) were purified from the peripheral blood of healthy donor (approval from Emory IRB000045690 "Phlebotomy of Healthy Adults for Research in Infectious Diseases and Immunology") by Ficoll-Paque Plus (GE Healthcare, Piscataway, NJ) density gradient centrifugation. CD4^+ ^T lymphocytes were isolated from PBMCs by depletion of non-CD4^+ ^cells (negative selection) using MACS CD4+ T Cell Isolation kit II (Miltenyi Biotec, Auburn, CA) and MACS LD columns (Miltenyi Biotec, Auburn, CA). Purified CD4^+ ^T cells were cultured in RPMI medium supplemented with 10% FBS, 10 ng/ml of Interleukin-2 (NIH AIDS Reagent Program), 2.5 μg/ml of phytohemagglutinin P (Sigma, St. Louis, MO) to activate the cells. Three days after cultivation, the purified cells were used for the assay.

The pCAGGS plasmids encoding full length or tail-truncated JRFL or HXB2 envelope glycoproteins (Env) were provided by Dr. J. Binley (Torrey Pines Institute, CA) [65]. The HIV-1 based packaging vector pR8ΔEnv lacking the *env *gene was from Dr. D. Trono (University of Geneva, Switzerland). HIV-1 BaL.01 Env expression vector (donated by Dr. J. Mascola [66]) and the pMM310 vector expressing BlaM-Vpr (donated by Dr. M. Miller [67] were obtained from the NIH AIDS Research and Reference Reagent Program. Vectors expressing MLV Gag-pol, Gag-GFP, and the MLV LTR lacZ [68] were provided by Dr. W. Mothes (Yale University).

The C52L recombinant peptide [27] was a gift from Dr. Min Lu (Cornell University). The BMS-806 compound [69,70] was synthesized by ChemPacific Corp. (Baltimore, MD), AMD3100 [71] and pronase were purchased from Sigma, and AD101 [72] was a gift from Dr. J. Strizki (Schering Plough, Kenilworth, NJ). Dynasore was purchased from Ascent Scientific LLC (Princeton, NJ), Calbiochem (Gibbstown, NJ), Santa Cruz Biothechnology (Santa Cruz, CA), Sigma (St. Louis, MO) and Tocris Bioscience (Ellisville, Missouri). Dynole 34-2 and dynole 31-2 (negaive control for dynole 34-2) were purchased from Ascent Scientific. DiD (1,1'-dioctadecyl-3,3,3',3'-tetramethylindodicarbocyanine, 4-chlorobenzenesulfonate salt), DiO (3,3'-dilinoleyloxacarbocyanine perchlorate) and R18 (octadecyl rhodamine B) were purchased from Invitrogen (Carlsbad, CA). Myristyltrimethylammonium Bromide (MiTMAB) was obtained from Calbiochem. Hanks' Balanced Salt Solution with calcium and magnesium (HBSS^++^) and poly-L-lysine were from Cellgro and Sigma, respectively.

### Plasmid construction

pMLV-Gag-mKO plasmid was constructed by modifying the pMLV-Gag-YFP plasmid [73]. The mKO sequence was amplified by PCR using KOD Xtreme DNA polymerase (Novagen, Gibbstown, NJ) and the forward and the reverse primers, 5'-TTGC*GGATCC*GGCGGCGGTGGAGCTAGCGTGAGTGTGATTAAACCAGAGATG -3' and 5'-TTCC*GCCGGC*TTAGATATCGGAATGAGCTACTGCATCTTCTAC-3', containing *BamHI *and *NaeI *restriction enzyme cleavage sites, respectively. The amplified fragment was cloned into pCR4 Blunt-TOPO vector (Invitrogen) and its sequence was verified. The YFP sequence in the pMLV-Gag-YFP was replaced with the mKO sequence from pCR4 Blunt-TOPO using *BamHI *and *NaeI *restriction sites.

### Virus production and characterization

To produce pseudoviruses containing the β-lactamase-Vpr (BlaM-Vpr) a 10 cm dish of 293T/17 cells were transfected with 2 μg pR8ΔEnv, 2 μg pMM310 vector expressing BlaM-Vpr, 1 μg pcRev [74], and 3 μg pCAGGS encoding JRFL or HXB2 or the BaL.01 expression vector, using PolyFect Transfection reagent (Qiagen, Valencia, CA). Fluorescently labeled pseudoviruses were produced by the PolyFect transfection of 293T/17 cells with 2 μg MLV-Gag-pol, 1 μg MLV-Gag-GFP or MLV-Gag-mKO, and 3 μg of the cytoplasmic tail-truncated HXB2 Env or JRFL Env provided by Dr. James Binley [65]. Twenty four hours post-transfection, cells were labeled with either 10 μM DiD, DiO or R18 for 4 h in the CO_2 _incubator at 37°C, as described in [26], washed and incubated for an additional 24 h in fresh growth media. Forty eight hours post-transfection virus-containing media was collected, passed through 0.45 μM filters, aliquoted and stored at -80°C. The infectious titer was determined by a β-Gal assay in TZM-bl cells [75].

### Virus-cell fusion assay

Measurements of HIV-1 fusion with target cells were carried out as described previously [23]. Briefly, viruses bearing the BlaM-Vpr chimera (MOI = 1) were bound to TZM-bl cells by centrifugation at 2095 × g, 4°C for 30 min. Unbound virus was washed off and fusion was initiated by shifting to 37°C and stopped after indicated times either by adding the respective inhibitors or by placing cells on ice (TB). Cells were then loaded with fluorescent CCF4-AM substrate (Invitrogen) and incubated overnight at 12°C. To measure HIV-1 fusion with suspension PM-1, CEMss or Jurkat cells or primary CD4^+ ^T cells were harvested by centrifugation (453 × g, 5 min at 4°C) and resuspended in HBSS^++ ^at 1·10^6 ^cells/ml. Aliquots of 1·10^5 ^cells/well were added to a poly-L-lysine-coated 96-well strip plate (Corning Costar) and cells were allowed to attach for 30 min at room temperature. Unbound cells were removed by washing with HBSS^++^, and the plates were blocked with 100 μl/well of HBSS^++^/10% FBS for 15 min at room temperature. Viruses bearing the BlaM-Vpr were bound and the fusion was initiated as described above. Intracellular β-lactamase activity (ratio of blue to green fluorescence) was measured using the Synergy HT fluorescence microplate reader (Bio-Tek, Germany).

To create the temperature-arrested stage (TAS) pseudoviruses (MOI = 0.8) were bound to TZM-bl cells by spinoculation at 2095 × g for 30 min at 4°C. Unbound virus was washed off, and the cells were incubated either at 22-24°C (HXB2 pseudoviruses), at 18-21°C (JRFL pseudoviruses) or at 4°C for 2.5 h. A high concentration (10 μM) of inhibitors of CD4 binding (BMS-806), and CXCR4 or CCR5 binding (AMD3100 and AD101, respectively) or of gp41 6-helix-bundle formation (C52L, 1 μM) were added either immediately prior to raising the temperature to 37°C (0 min) or after the indicated times. At the end of the 37°C incubation, cells were chilled and then loaded with fluorescent CCF2-AM substrate (Invitrogen) and incubated overnight at 12°C.

### Cell viability assay

Cytotoxic effects of dynamin inhibitors were evaluated by the MTS assay, using CellTiter 96 Aqueous reagent (Promega) according to the manufacturer's specifications. Briefly, 1·10^5 ^cells per well in a 96-well plate were treated with 50 μl DMEM containing specified compounds for 90 min at 37°C, after which time, DMEM was replaced with 50 μl DMEM/10% FBS containing 10 μl of CellTiter 96 Aqueous reagent. The cells were further incubated for additional 2 h at 37°C. The resulting absorbance from wells was recorded at 490 nm, using a plate reader.

### Diferential scanning calorimetry

Dynasore (Santa Cruz Biotechnology) was dissolved in a mixture of chloroform:methanol 2:1 to make a 1 mg/ml solution. A preparation of a dynasore monohydrate supplied by Sigma was found to have poorer solubility in non-polar solvents and showed somewhat poorer mixing with DiPoPE. Only the results from the Santa Cruz preparation are reported here. The dynamin inhibitor, MiTMAB, was dissolved in methanol so as to make a 1 mg/ml solution. Aliquots of these solutions were added to a set of tubes each containing 5 mg DiPoPE in chloroform:methanol 2:1, to end up with mole fractions of drug ranging from 0 to ~0.05. The solvent was evaporated under a stream of nitrogen gas so as to make thin films on the walls of the glass tubes. The tubes were further dried for three hours in a vacuum desiccator and kept under argon gas at -20°C until use. For calorimetry, the films were suspended in 0.8 ml PIPES buffer (20 mM PIPES, 1 mM EDTA, 140 mM NaCl, pH 7.4) with vigorous vortexing, degassed and added to the calorimeter cell at room temperature, with pure buffer in the reference cell. Temperature scans were made from 15 to 55°C at a 1°/min scan rate. The transition temperature for the liquid crystalline to the inverted hexagonal phase of DiPoPE is ~45°C. The transition temperature for each mixture was determined from a heating scan using a curve fitting program DA-2 supplied by Microcal, Inc. (Northampton, MA).

### Single virus imaging and image analysis

Fluorescence changes caused by dynasore and other compounds were monitored by adhering double-labeled viruses (~ 0.5·10^5 ^infectious units) for 30 min at 4°C to 8-well chamber slides (Lab-Tek) pre-coated with 0.1 mg/ml poly-L-lysine. Images were acquired every 10 sec for 10 min at room temperature, using the Personal DeltaVision imaging system (Applied Precision LLC., Issaquah, WA). Two consecutive images were acquired at each time point, by alternating the standard FITC, TRITC or CY5 excitation/emission filters, depending on the fluorescent probes used. Green and orange/red signals were separated by a quad (DAPI/FITC/TRITC/Cy5) dichroic beam-splitter and sequentially acquired using an EM-CCD camera (Photometrics).

For imaging HIV pseudovirus fusion, cells were grown overnight on 35mm Lab-Tek glass bottom dishes to approx. 90% confluency. Before imaging, cells were washed once with HBSS^++^, and pseudoviruses (MOI ~ 0.2) were spinoculated onto cells at 2095 × g for 30 min at 4°C. Free virus was removed by washing with cold HBSS^++^, cells were covered with 100 μl of cold HBSS^++ ^and transferred into an environmental enclosure maintained at 37°C. Once a suitable image field was found (within 1 min after transferring into the enclosure), 1 ml of pre-warmed HBSS^++ ^was added to the plate to initiate virus uptake and fusion, and image acquisition started. Images were acquired using the Personal DeltaVision imaging system using a UPlanFluo 40x/1.3NA oil-immersion objective (Olympus). Two consecutive images with 3 Z-sections 2.5 μm apart were acquired every 10 sec for 40 min by alternating the standard excitation/emission filters as listed above.

For confocal imaging of virus fusion, CEM.NKR-CCR5.Luc cells were attached to No. 0 glass coverslips coated with poly-L-lysine by a brief (20 min) centrifugation at 12°C, 600 × g, as described in [23]. Loosely bound cells were removed by washing, and coverslips were further centrifuged with the pseudovirus inoculum for 40 min at 12°C, 1000 × g. Cells were washed again and kept on ice for no longer than 3 h prior to imaging. Pieces of coverslip were then transferred into a custom-made glass-bottom imaging chamber, and virus fusion was initiated by raising the temperature locally to 37°C, using a custom-built temperature-jump setup [29]. Images were acquired with the C-Apo 40×/1.2NA water immersion objective, using the Zeiss LSM510Meta confocal microscope. Fluorescence signals from Gag-GFP and DiD labeled viruses were separated using a standard filter set. Three Z-sections separated by 3.0 μm were acquired at each time point with the pinhole set to 340 μm.

The HIV-1 pseudovirus fusion with TZM-bl cells was imaged as described previously [23], except that viruses were bound to cells by spinoculation at 4°C and additionally incubated for 2.5 h at 24°C to create TAS (or at 4°C in control experiments). Cells were kept on ice for up to 3 h and used for visualizing HIV-1 entry and fusion by confocal microscopy, as detailed above.

## List of Abbreviations

BlaM: beta-lactamase; C52L: HIV-1 gp41-derived inhibitory peptide; DiPoPE: dipalmitoleoyl phosphatidylethanolamine; MiTMAB: myristyltrimethylammonium bromide; TAS: temperature-arrested stage of HIV-1 fusion; TB: temperature block; T_H_: bilayer to hexagonal (H_II_) phase transition temperature.

## Competing interests

The authors declare that they have no competing interests.

## Authors' contributions

MD, MM, NK, KM, YK and RFE performed the experiments and analyzed the data. GBM conceived and planned the experiments and co-wrote the manuscript with MD and MM. RFE and RME co-wrote the manuscript and analyzed the data. All authors read and approved the final manuscript.

## Supplementary Material

Additional file 1 **Figure S1. Dynasore, but not dynole or MiTMAB, quenches the fluorescence of lipophilic dyes**. (A) HXB2 pseudotyped viruses co-labeled with Gag-mKO/DiD (upper panel) or Gag-mKO/DiO (lower panel, DiO is colored red) were adhered to poly-L-lysine coated coverslips. Sixty μM of dynasore (Santa Cruz) in HBSS^++ ^was added 1 min after beginning the image acquisition. Scale bar is 20 μm. (B) Mean fluorescence from co-labeled viruses (at least 100 particles per image field) was calculated and plotted over time. Dashed line shows the change in the DiD signal in another experiment when the compound was removed by washing. (C) HXB2 pseudoviruses co-labeled with Gag-GFP and R18 (C, D) or DiD (E-G) were adhered to poly-lysine coated chambered coverslips and imaged in HBSS^++ ^for 5 min at room temperature. Sixty μM of dynasore (C, D), 80 μM of MiTMAB (E, F) or 60 μM of dynole 34-2 (G) in HBSS^++ ^was added at the 1 min point (arrows). At least 100 co-labeled viruses were analyzed for changes in the mean fluorescence intensity of GFP, mKO, DiD and R18 over time. Click here for file

Additional file 2 **Figure S2. Transferrin uptake is blocked by dynasore and partially inhibited by dynole**. TZM-bl cells were left untreated (A, B) or were pretreated with 80 μM dynasore from different manufacturers (C-G), dynole 34-2 (H) or with dynole 31-2 (inactive control, I) dissolved in DMEM for 30 min at 4°C or 37°C. Cells were then incubated with 20 μg/ml of transferrin-Alexa488 (Invitrogen) in the cold, washed and further incubated for 10 min at 4°C (black histogram) or at 37°C (red line) in the absence or in the presence of dynamin inhibitors. Residual transferrin-Alexa488 at the cell surface was removed by pronase treatment (2 mg/ml, 10 min on ice), cells were washed with cold PBS supplemented with 10% FBS and resuspended in an appropriate volume of cold PBS. Transferring uptake was measured by flow cytometry (FACS LSRII, BD Biosciences) gating on live cells negative for the propidium iodide staining. Click here for file

Additional file 3 **Figure S3. Dynole, but not dynasore, affects the cell viability at concentrations that inhibit transferrin endocytosis**. (A) The dose-dependent effect of dynasore from different manufacturers on TZM-bl cell viability was as determined by the MTS assay, using CellTiter 96 Aqueous reagent (Promega) according to the manufacturer's specifications. The resulting absorbance measured at 490 nm in triplicate wells was normalized to the signal from untreated cells. Error bars are SEM. (B) The effect of dynole 34-2 and dynole 31-2 (inactive compound) from Ascent on TZM-bl cell viability determined by the MTS assay. Click here for file

Additional file 4 **Figure S4. The effect of dynole on the uptake of HIV-1 pseudoviruses**. Representative images of HXB2 pseudovirus uptake by TZM-bl cells are shown. Cells were pretreated with 60 μM dynole 34-2 (A) or with 60 μM dynole 31-2 (inactive compound) (B) in HBSS^++ ^for 30 min followed by binding of pseudoviruses co-labeled with HIV Gag-Cherry and EcpH-ICAM-1 (a chimera consisting of the Ecliptic pHluorin and the ICAM-1 transmembrane domain [23,25]). Cells were washed to remove unbound viruses and either imaged immediately (0 min) or incubated for 60 min at 37°C (60 min) in the presence of a dynamin inhibitor, using the Zeiss LSM780 confocal microscope. Scale bar is 20 μm. (C) Quantification of pseudovirus uptake exemplified in panels A and B. Z-stacks of cells from at least 5 random areas were acquired. Virus entry into acidic endosomes upon incubation at 37°C was manifested by quenching of the EcpH fluorescence (green) while the signal from mCherry-tagged viral cores (red) was not significantly altered. The total EcpH intensity from several hundreds of cell-associated double-labeled viruses was determined, and the ratio of the sum of EcpH signal to the sum of mCherry signal (normalized to that at time = 0) was plotted. Click here for file

Additional file 5 **Movie 1. Dynasore does not affect HXB2 virus motility**. HXB2 pseudoviruses co-labeled with Gag-mKO (green) and DiD (red) were bound to TZM-bl cells at 4°C, and cells were transferred to 37°C to initiate fusion, at which point the and imaging begun. After 5 min, 60 μM dynasore (Santa Cruz) was added to cells, and acquisition continued for an additional 15 min. Images were acquired using the Personal DeltaVision system. Click here for file

Additional file 6 **Movie 2. Rab5 motility is not altered by dynasore treatment**. TZM-bl cells were transfected with Rab5-GFP. Twenty four hours after transfection, cells were imaged at 37°C. After 5 min, 60 μM of dynasore (Santa Cruz) was introduced and imaging was continued for additional 15 min. Images were acquired using the Personal DeltaVision system and processed with the Spot Enhancing Filter 2D plugin from ImageJ. Click here for file

## References

[B1] Berger EA Murphy PM Farber JM Chemokine receptors as HIV-1 coreceptors: roles in viral entry, tropism, and disease Annu Rev Immunol 1999 17 657 700 10.1146/annurev.immunol.17.1.657 10358771

[B2] Doms RW Chemokine receptors and HIV entry Aids 2001 15 Suppl 1 S34 35 11403007 10.1097/00002030-200102001-00051

[B3] Gallo SA Finnegan CM Viard M Raviv Y Dimitrov A Rawat SS Puri A Durell S Blumenthal R The HIV Env-mediated fusion reaction Biochim Biophys Acta 2003 1614 36 50 10.1016/S0005-2736(03)00161-5 12873764

[B4] Melikyan GB Membrane fusion mediated by human immunodeficiency virus envelope glycoprotein Curr Top Membr 2011 68 81 106 21771496 10.1016/B978-0-12-385891-7.00004-0

[B5] Bedinger P Moriarty A von Borstel RC Donovan NJ Steimer KS Littman DR Internalization of the human immunodeficiency virus does not require the cytoplasmic domain of CD4 Nature 1988 334 162 165 10.1038/334162a0 3260353

[B6] Maddon PJ McDougal JS Clapham PR Dalgleish AG Jamal S Weiss RA Axel R HIV infection does not require endocytosis of its receptor, CD4 Cell 1988 54 865 874 10.1016/S0092-8674(88)91241-X 3261635

[B7] Stein BS Gowda SD Lifson JD Penhallow RC Bensch KG Engleman EG pH-independent HIV entry into CD4-positive T cells via virus envelope fusion to the plasma membrane Cell 1987 49 659 668 10.1016/0092-8674(87)90542-3 3107838

[B8] McClure MO Marsh M Weiss RA Human immunodeficiency virus infection of CD4-bearing cells occurs by a pH-independent mechanism Embo J 1988 7 513 518 3259178 10.1002/j.1460-2075.1988.tb02839.x PMC454348

[B9] Clavel F Charneau P Fusion from without directed by human immunodeficiency virus particles J Virol 1994 68 1179 1185 8289347 10.1128/jvi.68.2.1179-1185.1994 PMC236557

[B10] Rossio JL Esser MT Suryanarayana K Schneider DK Bess JW Vasquez GM Wiltrout TA Chertova E Grimes MK Sattentau Q Arthur LO Henderson LE Lifson JD Inactivation of human immunodeficiency virus type 1 infectivity with preservation of conformational and functional integrity of virion surface proteins J Virol 1998 72 7992 8001 9733838 10.1128/jvi.72.10.7992-8001.1998 PMC110135

[B11] Brandt SM Mariani R Holland AU Hope TJ Landau NR Association of chemokine-mediated block to HIV entry with coreceptor internalization J Biol Chem 2002 277 17291 17299 10.1074/jbc.M108232200 11782464

[B12] Pelchen-Matthews A Clapham P Marsh M Role of CD4 endocytosis in human immunodeficiency virus infection J Virol 1995 69 8164 8168 7494343 10.1128/jvi.69.12.8164-8168.1995 PMC189775

[B13] Aiken C Pseudotyping human immunodeficiency virus type 1 (HIV-1) by the glycoprotein of vesicular stomatitis virus targets HIV-1 entry to an endocytic pathway and suppresses both the requirement for Nef and the sensitivity to cyclosporin A J Virol 1997 71 5871 5877 9223476 10.1128/jvi.71.8.5871-5877.1997 PMC191842

[B14] Agosto LM Yu JJ Liszewski MK Baytop C Korokhov N Humeau LM O'Doherty U The CXCR4-tropic human immunodeficiency virus envelope promotes more-efficient gene delivery to resting CD4+ T cells than the vesicular stomatitis virus glycoprotein G envelope J Virol 2009 83 8153 8162 10.1128/JVI.00220-09 19493998 PMC2715791

[B15] Pace MJ Agosto L O'Doherty U R5 HIV Env and VSV-G Cooperate to Mediate Fusion to Naive CD4+T Cells J Virol 2010 10.1128/JVI.01851-10 PMC3014180 20980513

[B16] Yu D Wang W Yoder A Spear M Wu Y The HIV envelope but not VSV glycoprotein is capable of mediating HIV latent infection of resting CD4 T cells PLoS Pathog 2009 5 e1000633 10.1371/journal.ppat.1000633 19851458 PMC2760144

[B17] Garcia-Exposito L Barroso-Gonzalez J Puigdomenech I Machado JD Blanco J Valenzuela-Fernandez A HIV-1 requires Arf6-mediated membrane dynamics to efficiently enter and infect T lymphocytes Mol Biol Cell 2011 22 1148 1166 10.1091/mbc.E10-08-0722 21346189 PMC3078069

[B18] Fredericksen BL Wei BL Yao J Luo T Garcia JV Inhibition of endosomal/lysosomal degradation increases the infectivity of human immunodeficiency virus J Virol 2002 76 11440 11446 10.1128/JVI.76.22.11440-11446.2002 12388705 PMC136743

[B19] Schaeffer E Soros VB Greene WC Compensatory link between fusion and endocytosis of human immunodeficiency virus type 1 in human CD4 T lymphocytes J Virol 2004 78 1375 1383 10.1128/JVI.78.3.1375-1383.2004 14722292 PMC321368

[B20] Daecke J Fackler OT Dittmar MT Krausslich HG Involvement of clathrin-mediated endocytosis in human immunodeficiency virus type 1 entry J Virol 2005 79 1581 1594 10.1128/JVI.79.3.1581-1594.2005 15650184 PMC544101

[B21] von Kleist L Stahlschmidt W Bulut H Gromova K Puchkov D Robertson MJ MacGregor KA Tomlin N Pechstein A Chau N Chircop M Sakoff J von Kries JP Saenger W Krausslich HG Shupliakov O Robinson PJ McCluskey A Haucke V Role of the clathrin terminal domain in regulating coated pit dynamics revealed by small molecule inhibition Cell 2011 146 471 484 10.1016/j.cell.2011.06.025 21816279

[B22] Macia E Ehrlich M Massol R Boucrot E Brunner C Kirchhausen T Dynasore, a cell-permeable inhibitor of dynamin Dev Cell 2006 10 839 850 10.1016/j.devcel.2006.04.002 16740485

[B23] Miyauchi K Kim Y Latinovic O Morozov V Melikyan GB HIV enters cells via endocytosis and dynamin-dependent fusion with endosomes Cell 2009 137 433 444 10.1016/j.cell.2009.02.046 19410541 PMC2696170

[B24] Permanyer M Ballana E Este JA Endocytosis of HIV: anything goes Trends Microbiol 2010 18 543 551 10.1016/j.tim.2010.09.003 20965729

[B25] Miyauchi K Marin M Melikyan GB Visualization of retrovirus uptake and delivery into acidic endosomes Biochem J 2011 434 559 569 10.1042/BJ20101588 21175427 PMC3249399

[B26] Markosyan RM Cohen FS Melikyan GB Time-resolved imaging of HIV-1 Env-mediated lipid and content mixing between a single virion and cell membrane Mol Biol Cell 2005 16 5502 5513 10.1091/mbc.E05-06-0496 16195349 PMC1289397

[B27] Deng Y Zheng Q Ketas TJ Moore JP Lu M Protein design of a bacterially expressed HIV-1 gp41 fusion inhibitor Biochemistry 2007 46 4360 4369 10.1021/bi7001289 17371053

[B28] Chan DC Kim PS HIV entry and its inhibition Cell 1998 93 681 684 10.1016/S0092-8674(00)81430-0 9630213

[B29] Markosyan RM Cohen FS Melikyan GB HIV-1 envelope proteins complete their folding into six-helix bundles immediately after fusion pore formation Mol Biol Cell 2003 14 926 938 10.1091/mbc.E02-09-0573 12631714 PMC151570

[B30] Melikyan GB Markosyan RM Hemmati H Delmedico MK Lambert DM Cohen FS Evidence that the transition of HIV-1 gp41 into a six-helix bundle, not the bundle configuration, induces membrane fusion J Cell Biol 2000 151 413 424 10.1083/jcb.151.2.413 11038187 PMC2192659

[B31] Kliger Y Shai Y Inhibition of HIV-1 entry before gp41 folds into its fusion-active conformation J Mol Biol 2000 295 163 168 10.1006/jmbi.1999.3368 10623516

[B32] Cavrois M De Noronha C Greene WC A sensitive and specific enzyme-based assay detecting HIV-1 virion fusion in primary T lymphocytes Nat Biotechnol 2002 20 1151 1154 10.1038/nbt745 12355096

[B33] Bjorndal A Deng H Jansson M Fiore JR Colognesi C Karlsson A Albert J Scarlatti G Littman DR Fenyo EM Coreceptor usage of primary human immunodeficiency virus type 1 isolates varies according to biological phenotype J Virol 1997 71 7478 7487 9311827 10.1128/jvi.71.10.7478-7487.1997 PMC192094

[B34] Melikyan GB Barnard RJ Abrahamyan LG Mothes W Young JA Imaging individual retroviral fusion events: from hemifusion to pore formation and growth Proc Natl Acad Sci USA 2005 102 8728 8733 10.1073/pnas.0501864102 15937118 PMC1150829

[B35] Henderson HI Hope TJ The temperature arrested intermediate of virus-cell fusion is a functional step in HIV infection Virol J 2006 3 36 10.1186/1743-422X-3-36 16725045 PMC1482684

[B36] Mkrtchyan SR Markosyan RM Eadon MT Moore JP Melikyan GB Cohen FS Ternary complex formation of human immunodeficiency virus type 1 Env, CD4, and chemokine receptor captured as an intermediate of membrane fusion J Virol 2005 79 11161 11169 10.1128/JVI.79.17.11161-11169.2005 16103167 PMC1193594

[B37] Blumenthal R Clague MJ Durell SR Epand RM Membrane fusion Chem Rev 2003 103 53 69 10.1021/cr000036+ 12517181

[B38] Cheetham JJ Epand RM Andrews M Flanagan TD Cholesterol sulfate inhibits the fusion of Sendai virus to biological and model membranes J Biol Chem 1990 265 12404 12409 2165062

[B39] St.Vincent MR Colpitts CC Ustinov AV Muqadas M Joyce MA Barsby NA Epand R Epand R Khramyshev SS Valueva OA Korshun VA Tyrrell DLJ Schang LM Rigid Amphipathic Fusion Inhibitors, RAFIs, Small Molecule Antiviral Compounds Against Enveloped Viruses Proc Natl Acad Sci USA 2010 10.1073/pnas.1010026107 PMC2951442 20823220

[B40] Yeagle PL Young J Hui SW Epand RM On the mechanism of inhibition of viral and vesicle membrane fusion by carbobenzoxy-D-phenylalanyl-L-phenylalanylglycine Biochemistry 1992 31 3177 3183 10.1021/bi00127a019 1554703

[B41] Epand RM Lipid polymorphism and protein-lipid interactions Biochim Biophys Acta 1998 1376 353 368 9804988 10.1016/s0304-4157(98)00015-x

[B42] Epand RM Membrane lipid polymorphism: relationship to bilayer properties and protein function Methods Mol Biol 2007 400 15 26 10.1007/978-1-59745-519-0_2 17951724

[B43] Epand RM Role of Membrane Lipids in Modulating the Activity of Membrane-Bound Enzymes 2005 Boca Raton, FL: CRC Press

[B44] Epand RM Diacylglycerols, lysolecithin, or hydrocarbons markedly alter the bilayer to hexagonal phase transition temperature of phosphatidylethanolamines Biochemistry 1985 24 7092 7095 10.1021/bi00346a011 4084564

[B45] Quan A McGeachie AB Keating DJ van Dam EM Rusak J Chau N Malladi CS Chen C McCluskey A Cousin MA Robinson PJ Myristyl trimethyl ammonium bromide and octadecyl trimethyl ammonium bromide are surface-active small molecule dynamin inhibitors that block endocytosis mediated by dynamin I or dynamin II Mol Pharmacol 2007 72 1425 1439 10.1124/mol.107.034207 17702890

[B46] Epand RM Robinson KS Andrews ME Epand RF Dependence of the bilayer to hexagonal phase transition on amphiphile chain length Biochemistry 1989 28 9398 9402 10.1021/bi00450a022 2611238

[B47] Marsh M Helenius A Virus entry: open sesame Cell 2006 124 729 740 10.1016/j.cell.2006.02.007 16497584 PMC7112260

[B48] Mercer J Schelhaas M Helenius A Virus entry by endocytosis Annu Rev Biochem 2010 79 803 833 10.1146/annurev-biochem-060208-104626 20196649

[B49] Beer C Andersen DS Rojek A Pedersen L Caveola-dependent endocytic entry of amphotropic murine leukemia virus J Virol 2005 79 10776 10787 10.1128/JVI.79.16.10776-10787.2005 16051869 PMC1182675

[B50] Katen LJ Januszeski MM Anderson WF Hasenkrug KJ Evans LH Infectious entry by amphotropic as well as ecotropic murine leukemia viruses occurs through an endocytic pathway J Virol 2001 75 5018 5026 10.1128/JVI.75.11.5018-5026.2001 11333881 PMC114905

[B51] Kolokoltsov AA Deniger D Fleming EH Roberts NJ Karpilow JM Davey RA Small interfering RNA profiling reveals key role of clathrin-mediated endocytosis and early endosome formation for infection by respiratory syncytial virus J Virol 2007 81 7786 7800 10.1128/JVI.02780-06 17494077 PMC1933373

[B52] Guo J Wang W Yu D Wu Y Spinoculation triggers dynamic actin and cofilin activity that facilitates HIV-1 infection of transformed and resting CD4 T cells J Virol 2011 85 9824 9833 10.1128/JVI.05170-11 21795326 PMC3196392

[B53] Yoder A Yu D Dong L Iyer SR Xu X Kelly J Liu J Wang W Vorster PJ Agulto L Stephany DA Cooper JN Marsh JW Wu Y HIV envelope-CXCR4 signaling activates cofilin to overcome cortical actin restriction in resting CD4 T cells Cell 2008 134 782 792 10.1016/j.cell.2008.06.036 18775311 PMC2559857

[B54] Zhu P Liu J Bess J Chertova E Lifson JD Grise H Ofek GA Taylor KA Roux KH Distribution and three-dimensional structure of AIDS virus envelope spikes Nature 2006 441 847 852 10.1038/nature04817 16728975

[B55] Kruchten AE McNiven MA Dynamin as a mover and pincher during cell migration and invasion J Cell Sci 2006 119 1683 1690 10.1242/jcs.02963 16636070

[B56] Schroeder B Weller SG Chen J Billadeau D McNiven MA A Dyn2-CIN85 complex mediates degradative traffic of the EGFR by regulation of late endosomal budding EMBO J 2010 29 3039 3053 10.1038/emboj.2010.190 20711168 PMC2944065

[B57] Nicoziani P Vilhardt F Llorente A Hilout L Courtoy PJ Sandvig K van Deurs B Role for dynamin in late endosome dynamics and trafficking of the cation-independent mannose 6-phosphate receptor Mol Biol Cell 2000 11 481 495 10679008 10.1091/mbc.11.2.481 PMC14787

[B58] Robinet P Fradagrada A Monier MN Marchetti M Cogny A Moatti N Paul JL Vedie B Lamaze C Dynamin is involved in endolysosomal cholesterol delivery to the endoplasmic reticulum: role in cholesterol homeostasis Traffic 2006 7 811 823 10.1111/j.1600-0854.2006.00435.x 16787396

[B59] Lauvrak SU Torgersen ML Sandvig K Efficient endosome-to-Golgi transport of Shiga toxin is dependent on dynamin and clathrin J Cell Sci 2004 117 2321 2331 10.1242/jcs.01081 15126632

[B60] Engel S Heger T Mancini R Herzog F Kartenbeck J Hayer A Helenius A Role of endosomes in simian virus 40 entry and infection J Virol 2011 85 4198 4211 10.1128/JVI.02179-10 21345959 PMC3126231

[B61] Wei X Decker JM Liu H Zhang Z Arani RB Kilby JM Saag MS Wu X Shaw GM Kappes JC Emergence of resistant human immunodeficiency virus type 1 in patients receiving fusion inhibitor (T-20) monotherapy Antimicrob Agents Chemother 2002 46 1896 1905 10.1128/AAC.46.6.1896-1905.2002 12019106 PMC127242

[B62] Nara PL Fischinger PJ Quantitative infectivity assay for HIV-1 and-2 Nature 1988 332 469 470 10.1038/332469a0 3281026

[B63] Trkola A Matthews J Gordon C Ketas T Moore JP A cell line-based neutralization assay for primary human immunodeficiency virus type 1 isolates that use either the CCR5 or the CXCR4 coreceptor J Virol 1999 73 8966 8974 10516002 10.1128/jvi.73.11.8966-8974.1999 PMC112928

[B64] Lusso P Cocchi F Balotta C Markham PD Louie A Farci P Pal R Gallo RC Reitz MS Jr Growth of macrophage-tropic and primary human immunodeficiency virus type 1 (HIV-1) isolates in a unique CD4+ T-cell clone (PM1): failure to downregulate CD4 and to interfere with cell-line-tropic HIV-1 J Virol 1995 69 3712 3720 7745720 10.1128/jvi.69.6.3712-3720.1995 PMC189087

[B65] Binley JM Cayanan CS Wiley C Schulke N Olson WC Burton DR Redox-triggered infection by disulfide-shackled human immunodeficiency virus type 1 pseudovirions J Virol 2003 77 5678 5684 10.1128/JVI.77.10.5678-5684.2003 12719560 PMC154040

[B66] Li Y Svehla K Mathy NL Voss G Mascola JR Wyatt R Characterization of antibody responses elicited by human immunodeficiency virus type 1 primary isolate trimeric and monomeric envelope glycoproteins in selected adjuvants J Virol 2006 80 1414 1426 10.1128/JVI.80.3.1414-1426.2006 16415019 PMC1346938

[B67] Tobiume M Lineberger JE Lundquist CA Miller MD Aiken C Nef does not affect the efficiency of human immunodeficiency virus type 1 fusion with target cells J Virol 2003 77 10645 10650 10.1128/JVI.77.19.10645-10650.2003 12970449 PMC228506

[B68] Sherer NM Lehmann MJ Jimenez-Soto LF Ingmundson A Horner SM Cicchetti G Allen PG Pypaert M Cunningham JM Mothes W Visualization of retroviral replication in living cells reveals budding into multivesicular bodies Traffic 2003 4 785 801 10.1034/j.1600-0854.2003.00135.x 14617360

[B69] Lin PF Blair W Wang T Spicer T Guo Q Zhou N Gong YF Wang HG Rose R Yamanaka G Robinson B Li CB Fridell R Deminie C Demers G Yang Z Zadjura L Meanwell N Colonno R A small molecule HIV-1 inhibitor that targets the HIV-1 envelope and inhibits CD4 receptor binding Proc Natl Acad Sci USA 2003 100 11013 11018 10.1073/pnas.1832214100 12930892 PMC196918

[B70] Si Z Madani N Cox JM Chruma JJ Klein JC Schon A Phan N Wang L Biorn AC Cocklin S Chaiken I Freire E Smith AB Sodroski JG Small-molecule inhibitors of HIV-1 entry block receptor-induced conformational changes in the viral envelope glycoproteins Proc Natl Acad Sci USA 2004 101 5036 5041 10.1073/pnas.0307953101 15051887 PMC387369

[B71] Donzella GA Schols D Lin SW Este JA Nagashima KA Maddon PJ Allaway GP Sakmar TP Henson G De Clercq E Moore JP AMD3100, a small molecule inhibitor of HIV-1 entry via the CXCR4 co-receptor Nat Med 1998 4 72 77 10.1038/nm0198-072 9427609

[B72] Trkola A Kuhmann SE Strizki JM Maxwell E Ketas T Morgan T Pugach P Xu S Wojcik L Tagat J Palani A Shapiro S Clader JW McCombie S Reyes GR Baroudy BM Moore JP HIV-1 escape from a small molecule, CCR5-specific entry inhibitor does not involve CXCR4 use Proc Natl Acad Sci USA 2002 99 395 400 10.1073/pnas.012519099 11782552 PMC117571

[B73] Andrawiss M Takeuchi Y Hewlett L Collins M Murine leukemia virus particle assembly quantitated by fluorescence microscopy: role of Gag-Gag interactions and membrane association J Virol 2003 77 11651 11660 10.1128/JVI.77.21.11651-11660.2003 14557651 PMC229285

[B74] Malim MH Hauber J Fenrick R Cullen BR Immunodeficiency virus rev trans-activator modulates the expression of the viral regulatory genes Nature 1988 335 181 183 10.1038/335181a0 3412474

[B75] Kimpton J Emerman M Detection of replication-competent and pseudotyped human immunodeficiency virus with a sensitive cell line on the basis of activation of an integrated beta-galactosidase gene J Virol 1992 66 2232 2239 1548759 10.1128/jvi.66.4.2232-2239.1992 PMC289016

