# Supplementary figures and images for: Inhibition of HIV-1 endocytosis allows lipid mixing at the plasma membrane, but not complete fusion
Source: Retrovirology. 2011 Dec 6;8:99. doi: 10.1186/1742-4690-8-99 (PMC3297528; doi:10.1186/1742-4690-8-99)

**A**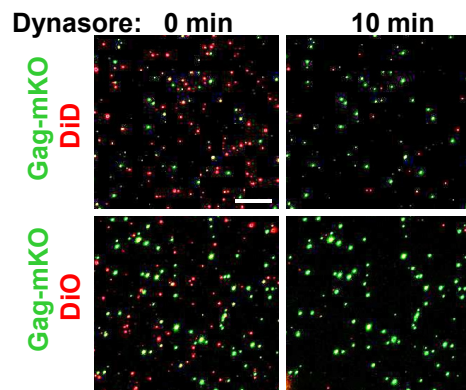**B**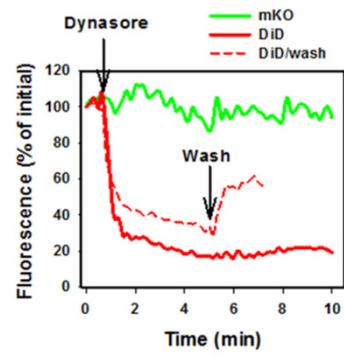**C**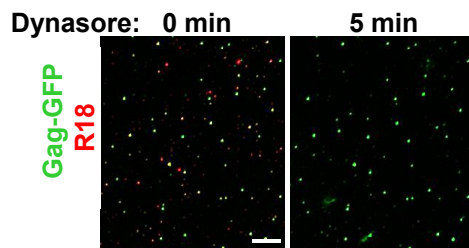**D**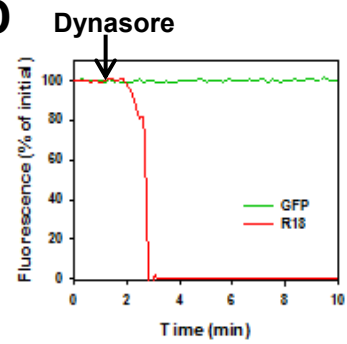**E**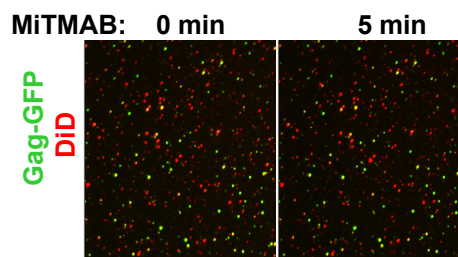**F**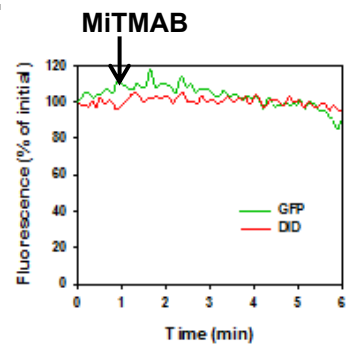**G**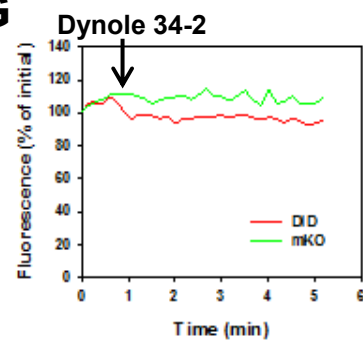

Supplement: Additional file 1 — Figure S1. Dynasore, but not dynole or MiTMAB, quenches the fluorescence of lipophilic dyes. (A) HXB2 pseudotyped viruses co-labeled with Gag-mKO/DiD (upper panel) or Gag-mKO/DiO (lower panel, DiO is colored red) were adhered to poly-L-lysine coated coverslips. Sixty μM of dynasore (Santa Cruz) in HBSS++ was added 1 min after beginning the image acquisition. Scale bar is 20 μm. (B) Mean fluorescence from co-labeled viruses (at least 100 particles per image field) was calculated and plotted over time. Dashed line shows the change in the DiD signal in another experiment when the compound was removed by washing. (C) HXB2 pseudoviruses co-labeled with Gag-GFP and R18 (C, D) or DiD (E-G) were adhered to poly-lysine coated chambered coverslips and imaged in HBSS++ for 5 min at room temperature. Sixty μM of dynasore (C, D), 80 μM of MiTMAB (E, F) or 60 μM of dynole 34-2 (G) in HBSS++ was added at the 1 min point (arrows). At least 100 co-labeled viruses were analyzed for changes in the mean fluorescence intensity of GFP, mKO, DiD and R18 over time. [file 1742-4690-8-99-S1.PDF]

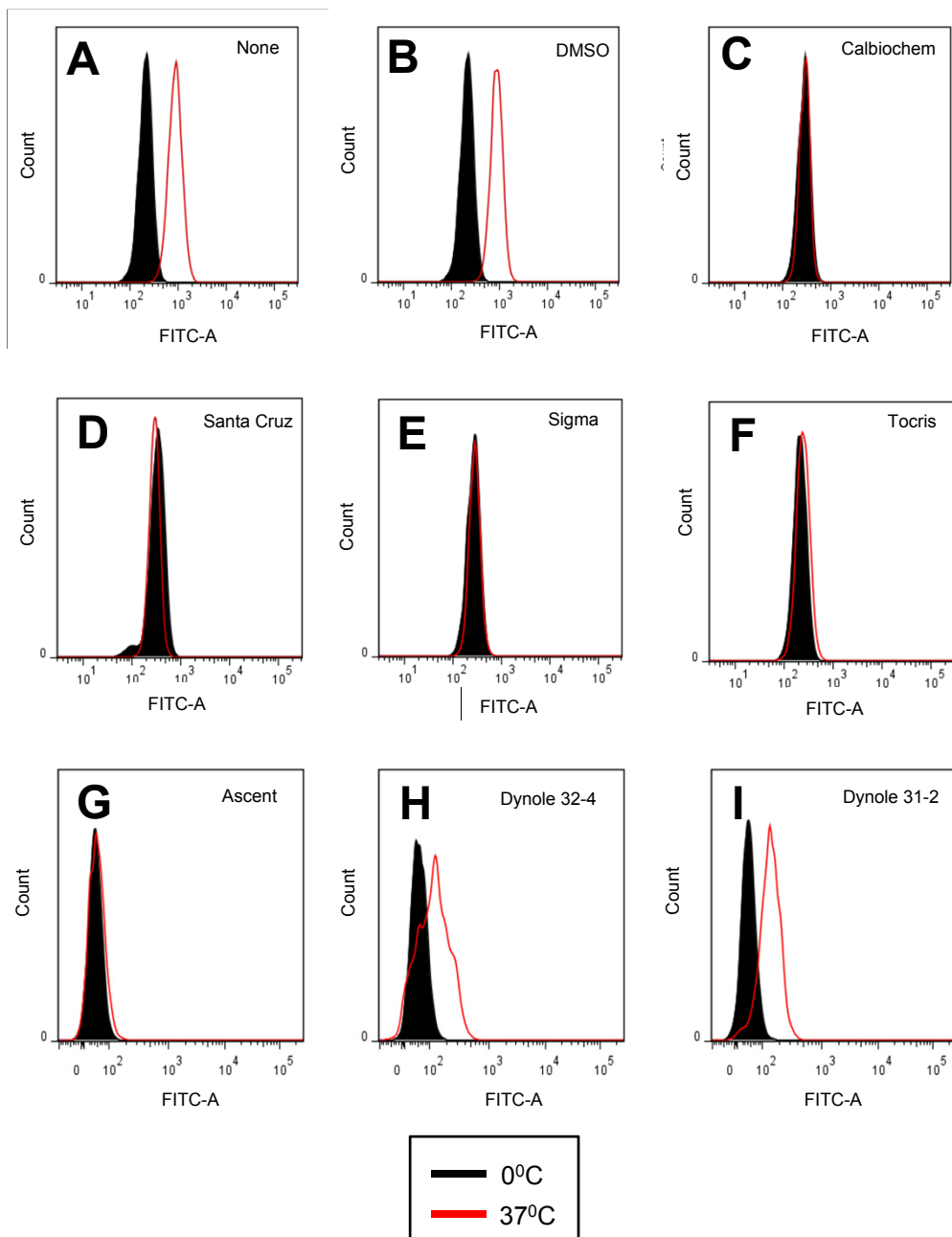

Supplement: Additional file 2 — Figure S2. Transferrin uptake is blocked by dynasore and partially inhibited by dynole. TZM-bl cells were left untreated (A, B) or were pretreated with 80 μM dynasore from different manufacturers (C-G), dynole 34-2 (H) or with dynole 31-2 (inactive control, I) dissolved in DMEM for 30 min at 4°C or 37°C. Cells were then incubated with 20 μg/ml of transferrin-Alexa488 (Invitrogen) in the cold, washed and further incubated for 10 min at 4°C (black histogram) or at 37°C (red line) in the absence or in the presence of dynamin inhibitors. Residual transferrin-Alexa488 at the cell surface was removed by pronase treatment (2 mg/ml, 10 min on ice), cells were washed with cold PBS supplemented with 10% FBS and resuspended in an appropriate volume of cold PBS. Transferring uptake was measured by flow cytometry (FACS LSRII, BD Biosciences) gating on live cells negative for the propidium iodide staining. [file 1742-4690-8-99-S2.PDF]

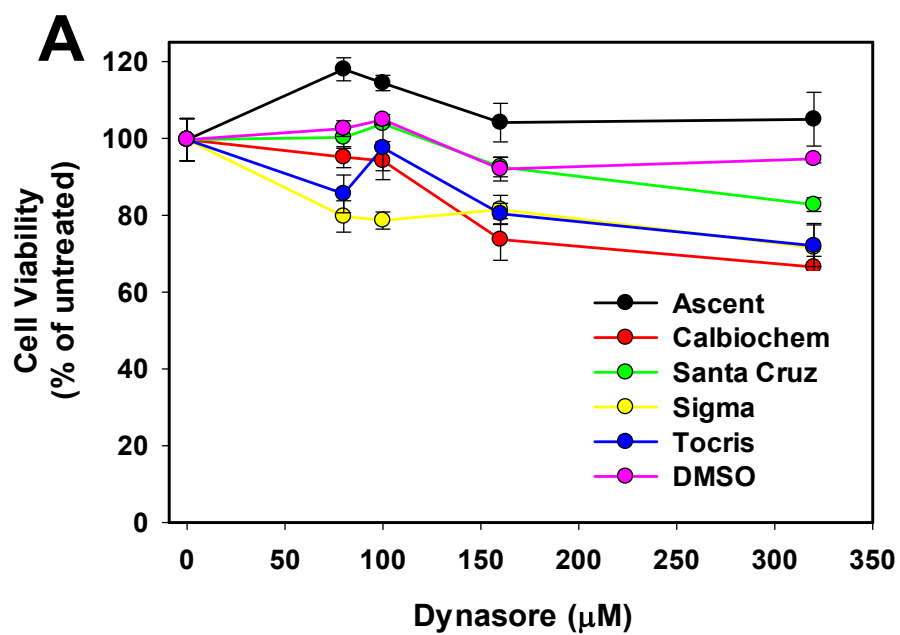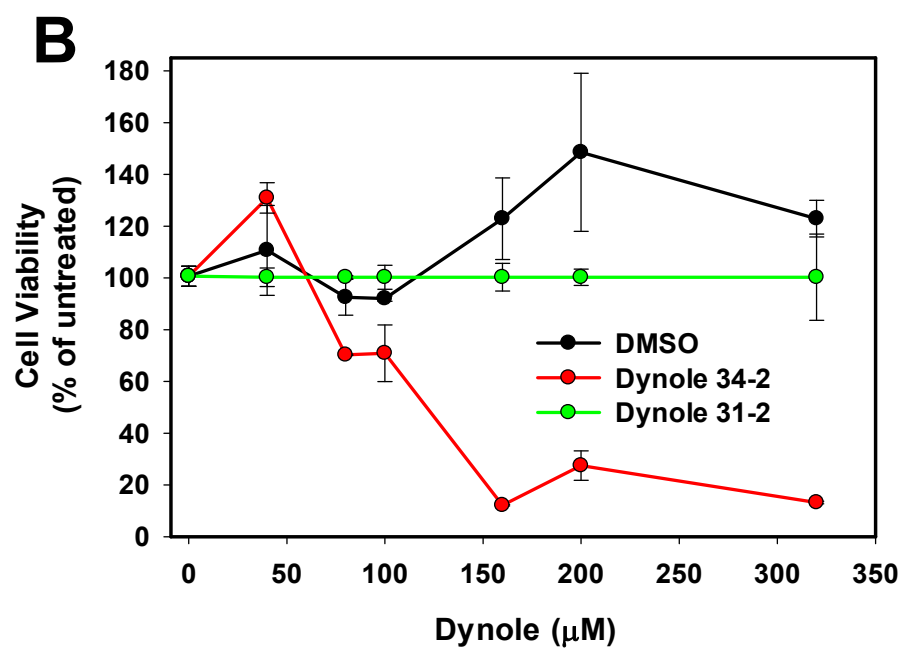

Supplement: Additional file 3 — Figure S3. Dynole, but not dynasore, affects the cell viability at concentrations that inhibit transferrin endocytosis. (A) The dose-dependent effect of dynasore from different manufacturers on TZM-bl cell viability was as determined by the MTS assay, using CellTiter 96 Aqueous reagent (Promega) according to the manufacturer's specifications. The resulting absorbance measured at 490 nm in triplicate wells was normalized to the signal from untreated cells. Error bars are SEM. (B) The effect of dynole 34-2 and dynole 31-2 (inactive compound) from Ascent on TZM-bl cell viability determined by the MTS assay. [file 1742-4690-8-99-S3.PDF]

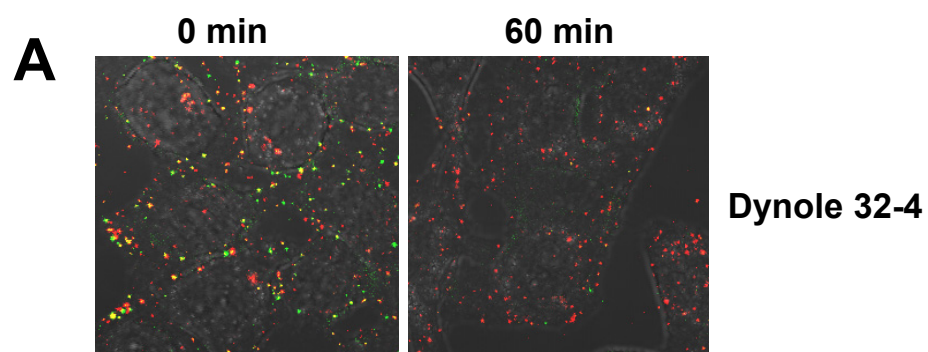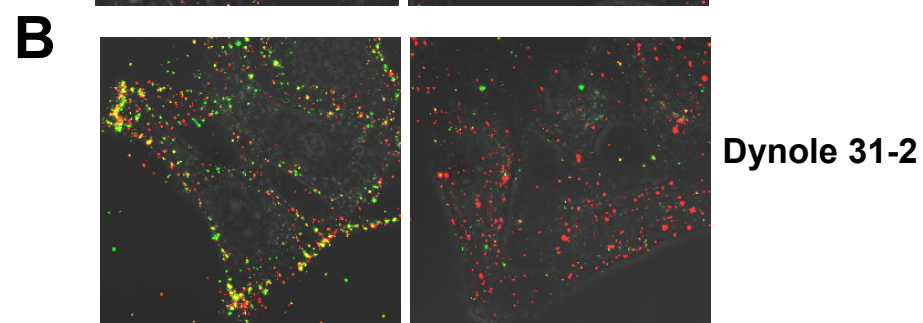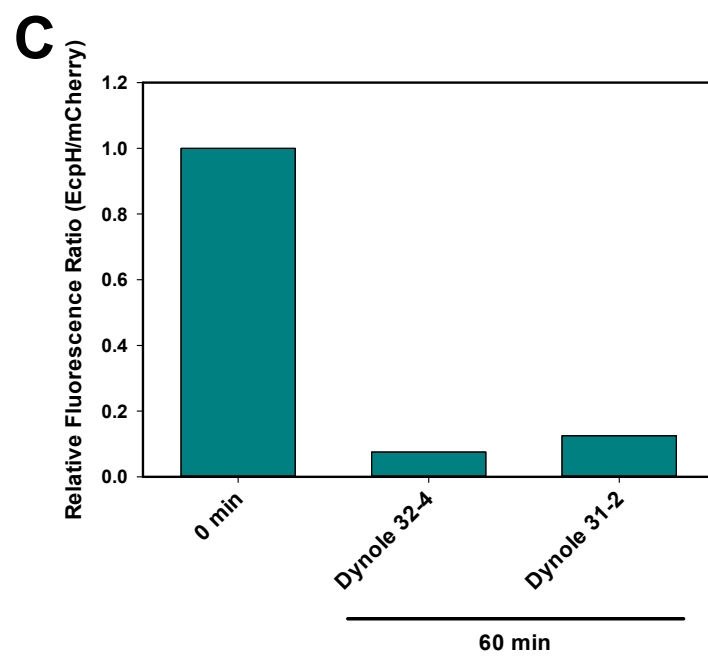

Supplement: Additional file 4 — Figure S4. The effect of dynole on the uptake of HIV-1 pseudoviruses. Representative images of HXB2 pseudovirus uptake by TZM-bl cells are shown. Cells were pretreated with 60 μM dynole 34-2 (A) or with 60 μM dynole 31-2 (inactive compound) (B) in HBSS++ for 30 min followed by binding of pseudoviruses co-labeled with HIV Gag-Cherry and EcpH-ICAM-1 (a chimera consisting of the Ecliptic pHluorin and the ICAM-1 transmembrane domain [23,25]). Cells were washed to remove unbound viruses and either imaged immediately (0 min) or incubated for 60 min at 37°C (60 min) in the presence of a dynamin inhibitor, using the Zeiss LSM780 confocal microscope. Scale bar is 20 μm. (C) Quantification of pseudovirus uptake exemplified in panels A and B. Z-stacks of cells from at least 5 random areas were acquired. Virus entry into acidic endosomes upon incubation at 37°C was manifested by quenching of the EcpH fluorescence (green) while the signal from mCherry-tagged viral cores (red) was not significantly altered. The total EcpH intensity from several hundreds of cell-associated double-labeled viruses was determined, and the ratio of the sum of EcpH signal to the sum of mCherry signal (normalized to that at time = 0) was plotted. [file 1742-4690-8-99-S4.PDF]
